# Triarylamine-Modified Phenothiazine Small Molecules as Hole-Transporting Materials in Wide-Band-Gap Perovskite Solar Cells

**DOI:** 10.1021/acsami.6c03041

**Published:** 2026-06-09

**Authors:** Daniel Augusto Machado de Alencar, Jessica Barichello, Raffaele Borrelli, Pierluigi Quagliotto, Francesca Brunetti, Matteo Bonomo, Fabio Matteocci, Aldo di Carlo, Claudia Barolo

**Affiliations:** † Department of Chemistry and NIS Interdepartmental Center, 9314 University of Turin, 10135 Turin, Italy; ‡ CNR-ISM, 204549 Istituto di Struttura della Materia, Consiglio Nazionale delle Ricerche, 00133 Rome, Italy; § Department of Agricultural, Forest and Food Sciences, University of Turin, Grugliasco, 10095 Turin, Italy; ∥ CHOSE, Department of Electronic Engineering, University of Rome Tor Vergata, 00133 Rome, Italy; ⊥ Department of Basic and Applied Sciences for Engineering (SBAI), 9311 Sapienza University of Rome, 00161 Rome, Italy; # National Interuniversity Consortium of Materials Science and Technology (INSTM), 50121 Florence, Italy; ¶ Istituto di Scienza, Tecnologia e Sostenibilità per lo sviluppo dei Materiali Ceramici (ISSMC−CNR), 48018 Faenza, Italy

**Keywords:** hole-transporting materials, phenothiazine, perovskite solar cells, indoor photovoltaics, sustainability, semitransparent devices

## Abstract

Perovskite solar cells (PSCs) offer exceptional tunability of optoelectronic properties, enabling wide-band-gap absorbers that are highly attractive for semitransparent devices in building-integrated photovoltaics (BIPV). However, challenges associated with stability, scalability, and materials’ cost continue to limit their practical deployment, highlighting the pivotal role of hole transport materials (HTMs) in achieving high efficiency and durable device operation. Herein, we report the rational design and synthesis of three novel small-molecule HTMs based on phenothiazine–triarylamine cores, prepared via concise synthetic routes with moderate-to-high yields. The electron-rich, nonplanar phenothiazine scaffold enables suppressed aggregation and favorable energy-level alignment, rendering these materials particularly suitable for wide-band-gap and semitransparent PSCs. When implemented in FAPbBr_3_-based semitransparent devices, two candidates (**SM1** and **SM2**) achieve power conversion efficiencies comparable to those of the state-of-the-art poly­(triarylamine) (PTAA) (PCE = 6.26% and 6.09% for **SM1** and **SM2**, respectively, vs 6.39% for PTAA). Notably, their enhanced optical transparency leads to comparable light-utilization efficiency (LUE) (4.05 and 3.99 for **SM1** and **SM2**, respectively, vs 4.07 for PTAA), with outstanding and superior bifaciality factors (84% and 82% for **SM1** and **SM2**, respectively, vs 81% for PTAA), providing a distinct advantage beyond conventional opaque-PV efficiency metrics. These findings position phenothiazine-based HTMs as promising, cost-effective alternatives to PTAA for scalable semitransparent perovskite solar cells.

## Graphical abstract

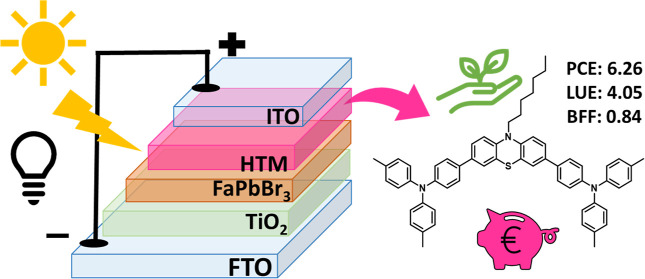

## Introduction

Since the pioneering work of Miyasaka in 2009,^1^ introducing perovskites as a light-harvesting material in dye-sensitized solar cells, research on perovskite solar cells (PSCs) has expanded at an exceptional pace. The power conversion efficiency (PCE) has progressed from 3.9% to 10% in solid-state configurations^2,3^ and has more recently approached a remarkable 27.0%.^4–6^ Not only that, PSCs offer significant potential beyond their outstanding efficiencies. Indeed, the characteristic crystal structure of perovskites (ABX_3_, where A is an organic or inorganic cation, B is a metal such as lead, and X is a halide) enables a tunable band gap by formulation engineering, allowing, in turn, the fabrication of wide-band-gap perovskites that are semitransparent and nearly colorless. This band gap tunability broadens the scope of photovoltaic (PV) applications, enabling integration into building-integrated photovoltaics (BIPV), agrivoltaics, and indoor energy-harvesting systems without compromising environmental compatibility.^7,8^ Among wide-band-gap candidates, formamidinium lead bromide (FAPbBr_3_) has emerged as particularly promising, exhibiting a band gap of approximately 2.3 eV, with a valence band maximum around −5.6 eV and a conduction band minimum near −3.3 eV, which are well suited for semitransparent photovoltaic applications.^9–12^ To capitalize on this semitransparency, recent developments have incorporated transparent counter electrodes (TCOs) into fully transparent device stacks, achieving an Average Visible Transmittance (AVT) of up to 70% and a Light Utilization Efficiency (LUE)^13^ of 5.72 in an n-i-p architecture.^9^ Moreover, such a device structure could also exploit the bifacial nature of PSCs, wherein photons arriving on both the front and rear surfaces contribute to photocurrent generation. For FaPbBr_3_, the bifaciality factor has been reported to reach values as high as 87%.^9^ In parallel, inverted device architectures employing ultrathin or monolayer hole-extraction layers have also been explored for wide-band-gap (∼2.2–2.3 eV) perovskites such as FAPbBr_3_, demonstrating promising efficiencies while minimizing parasitic absorption losses.^14^ However, these approaches often rely on highly controlled interfacial engineering and may present challenges in terms of process reproducibility and large-area scalability. Therefore, the development of solution-processable hole transport materials (HTMs) compatible with more conventional device architectures remains highly relevant for practical applications.

Besides active materials, HTMs are also critical components in the development of see-through but high-performance and long-term stable perovskite solar cells (PSCs). The incorporation of an HTM is indispensable in PSC architectures, as it facilitates the efficient extraction and transport of photogenerated holes from the perovskite active layer to the metal electrode.^15^ In addition, the HTM serves a protective function by mitigating metal diffusion from commonly employed electrodes such as Au, Ag, and Al into the perovskite layer.^16,17^ Recently, the request for highly efficient and transparent semiconductors has attracted considerable attention, particularly with respect to the exponential interest in the next generation of tandem solar cells.^18,19^ A number of inorganic HTMs exhibit intrinsic (if deposited as a thin film) transparency, including CuSCN,^20–22^ CuI,^23^ NiO_*x*_,^24,25^ MoO_*x*_,^26^ CuGaO_2_,^27^ and Sr_3_Cu_2_Sc_2_O_5_S_2_.^28^ While several inorganic HTMs have demonstrated promising performance, organic HTMs offer the advantage of tunable physicochemical properties. Currently, the most widely used organic HTMs are Spiro-OMeTAD and poly­(triarylamine) (PTAA).^29–31^ However, both materials face limitations with respect to large-scale commercialization due to their high synthesis and processing costs.^32,33^ Recent studies have increasingly focused on the development of dopant-free small-molecule HTMs as cost-effective alternatives to conventional materials such as Spiro-OMeTAD. In particular, molecular engineering strategies based on donor–acceptor architectures and enhanced intramolecular charge transfer have been shown to improve hole mobility, energy-level alignment, and device stability, leading to significant gains in current density and overall efficiency.^34–36^ Consequently, the design and synthesis of efficient, low-cost, transparent alternatives are imperative to further advance PSC performance and accelerate their market deployment.^37^

The development of transparent organic HTMs presents significant challenges. First, extending the π-conjugation of a delocalized system essentially minimizes reorganization energy (λ) and enhances electronic coupling (V) among adjacent molecules in the solid-state packing structure, resulting in a red shift (bathochromic shift) of the absorption spectrum.^38^ Furthermore, precise alignment of the HTM highest occupied molecular orbital (HOMO) with the perovskite valence band is critical for maximizing device efficiency,^39^ yet such tuning must be achieved ideally without compromising optical transparency. Therefore, an ideal HTM should simultaneously meet multiple criteria: (i) appropriate energy-level alignment, (ii) high hole mobility, (iii) good solubility in processing solvents, (iv) hydrophobicity, (v) low cost, (vi) thermal and light stability, and, in the case of transparent PSC architectures, (vii) optical transparency.^40^ To this end, various heterocyclic frameworks have been explored as building blocks for small-molecule HTMs, including benzodithiophene, carbazole triarylamine, truxene, azatruxene, benzothiadiazole, and diketopyrrolopyrrole, among others.^41,42^ In the pursuit of novel HTMs, phenothiazine (PTZ) has emerged as a highly promising scaffold for molecular design and synthesis. PTZ is an attractive candidate owing to its electron-rich and structurally flexible heterocycle, its low cost and commercial availability as a starting material, its wide processability in diverse, more environmentally friendly organic solvents, and the facile tunability of its redox properties.^43^ As a matter of fact, the presence of electron-donating nitrogen and sulfur atoms renders PTZ particularly suitable for chemical engineering toward donor–acceptor (D–A) systems with enhanced intramolecular charge-transfer (ICT) characteristics.^44–47^ Furthermore, the intrinsic nonplanar geometry of PTZ, characterized by a dihedral angle of 158.5° between the planes of its two benzene rings, mitigates intermolecular excimer formation and limits molecular aggregation.^47–49^ Structural tunability is further enhanced by the possibility of functionalization at the N-10, C-2/C-8, and C-3/C-7 positions, offering broad opportunities for tailoring electronic and physicochemical properties. Importantly, the conjugated backbone plays a dominant role in determining the electronic structure of organic semiconductors, particularly with respect to the band gap and frontier energy levels.^49^

Phenothiazine-based HTMs have demonstrated considerable potential as viable sustainable alternatives to other small-molecule HTMs like Spiro-OMeTAD and even polymeric HTMs like PTAA.^50^ In the present work, we report the preparation of three novel HTMs derived from the PTZ scaffold. The molecular designs were guided by our previous studies, in which a series of phenothiazine–triphenylamine copolymers bearing diverse functional groups were developed.^51^ The principal structural modifications in the current series involve functionalization of the phenothiazine nitrogen with either an alkyl substituent or an aromatic moiety, as well as oxidation of the sulfur atom within the phenothiazine core. These materials were subsequently assessed as HTMs in wide-band-gap PSCs, demonstrating efficiencies comparable to PTAA alongside enhanced optical transmission, highlighting their strong potential to exceed state- of-the-art HTMs from a light-utilization efficiency standpoint.

## Result and Discussion

### Synthesis

The synthetic routes for **SM1**, **SM2**, and **SM3** are illustrated in Scheme S1. The two phenothiazine cores, **ii** and **iv**, were synthesized in two steps starting from commercially available phenothiazine, whereas core **v** was accessed via oxidation of **iv**. To prepare the **ii/iv** intermediates, the alkyl or aromatic side chains were introduced at the phenothiazine nitrogen atom through nucleophilic substitution—using 1-iodohexane for **i** and via the Buchwald–Hartwig protocol for **iii**. The resulting scaffolds were subsequently converted into the corresponding brominated derivatives using liquid bromine in chloroform. To further modulate the frontier molecular orbital energy levels of the novel HTMs, **iv** was oxidized with *meta*-chloroperbenzoic acid (mCPBA) in dichloromethane at 0 °C, affording 10-(4-butoxyphenyl)-10H-phenothiazine 5-oxide (**v**) in 88% yield. Finally, the three HTMs, **SM1–SM3**, were synthesized via Suzuki–Miyaura C–C cross-coupling between the intermediates (**ii**, **iv**, and **v**) and intermediate **ix**, providing the target HTMs in yields ranging from moderate to excellent.

### Characterization

All the intermediates and the products were fully characterized by ^1^H and ^13^C NMR; for the final materials only, FTIR and mass spectrometry were also conducted (Figures S1–S13—see Supporting Information) (see Figure 1).

**Figure 1:**
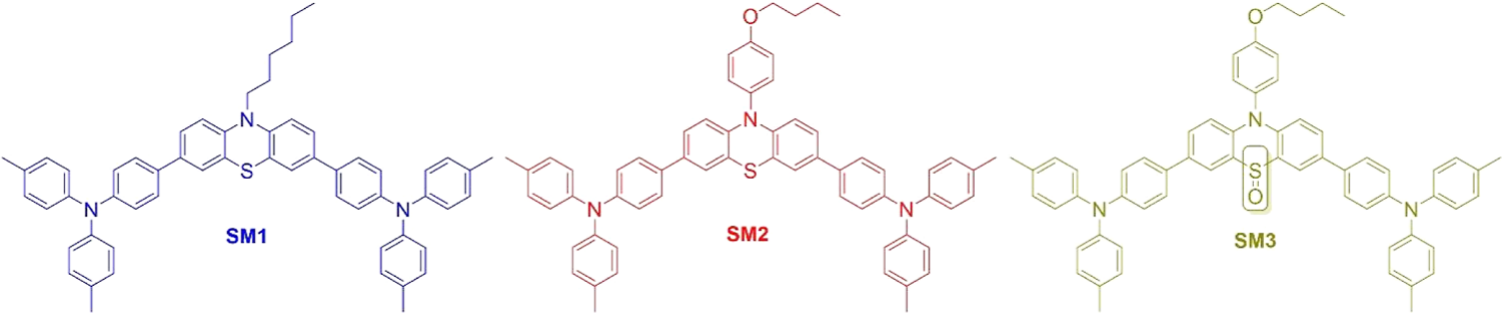
Structure of the PTZ-based transparent HTMs.

The glass transition temperature (*T*_g_) and degradation temperature (*T*_d_) are critical parameters for evaluating the thermal stability of organic molecules intended for use as HTMs in PSCs. Indeed, one of the main factors contributing to rapid performance loss in PSCs is the transition of metastable HTM amorphous phases under the heat generated during device operation.^52^ Thermogravimetric analysis (TGA, Figure 2c) demonstrated that all three materials exhibit excellent thermal stability, remaining intact beyond 300 °C (Table 1). Such robustness ensures compatibility with the processing conditions of PSCs and supports their potential use also in inverted device architectures.

**Figure 2:**
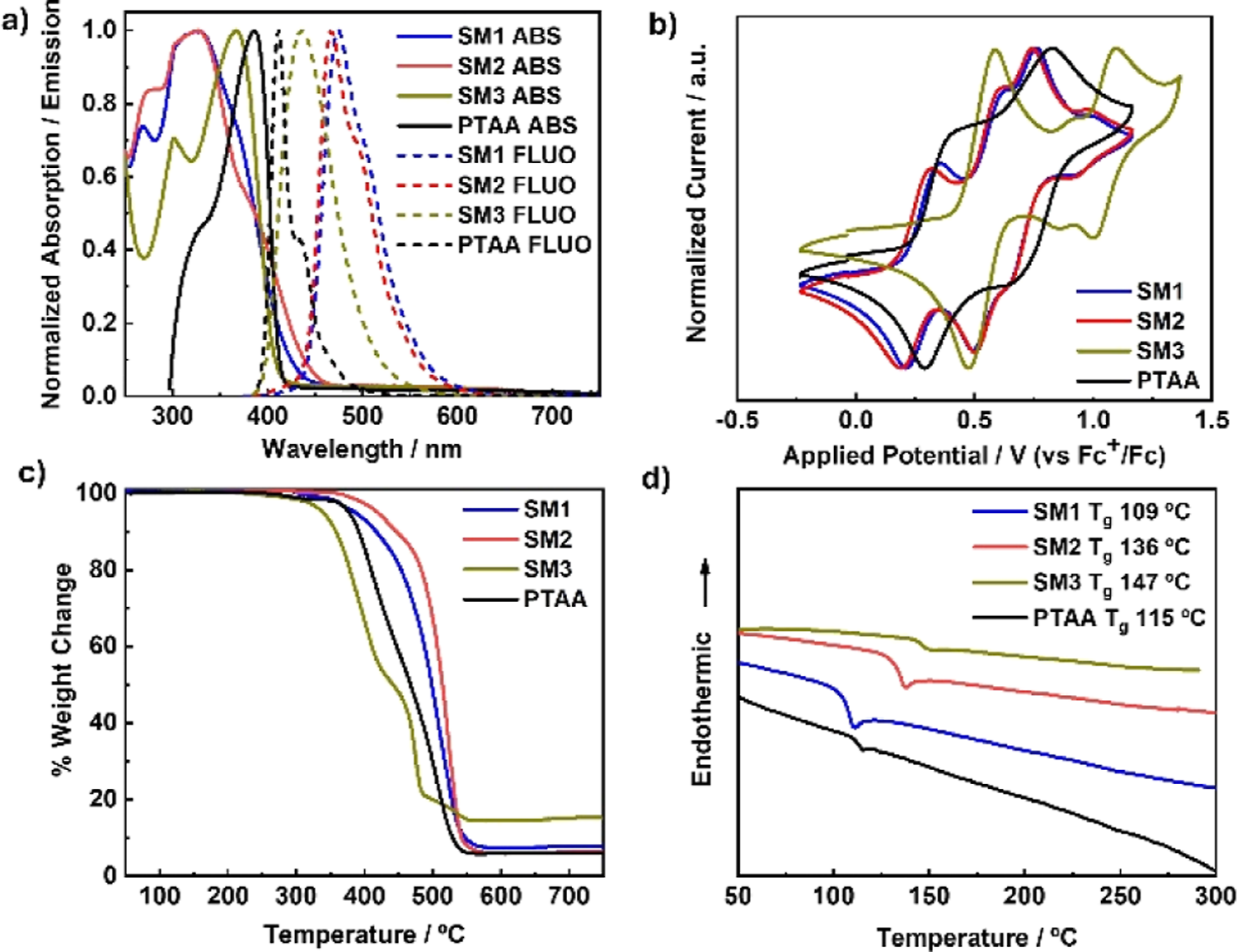
(a) Normalized absorption/emission spectra of the HTMs (2.5 μM in DCM), (b) cyclic voltammetry of HTMs in DCM solutions using Bu_4_NBF_4_ (0.1 M) as the supporting electrode, and (c) thermogravimetric analysis of HTMs measured in air. (d) Second DSC heating traces of HTMs in nitrogen.

**Table 1:** Summary of Characterization of **SM1-3**

	λ_ABS_ (nm)	λ_EMI_ (nm)	E_g_ (eV)	HOMO (eV)	LUMO (eV)	*T*_D_ (°C)	*T*_g_ (°C)
**SM1**	360	475	2.94	–5.38	–2.44	387	109
**SM2**	370	468	2.78	–5.35	–2.57	419	136
**SM3**	367	436	3.05	–5.63	–2.58	339	147
**PTAA**	386	378	2.86	–5.14	–2.18	377	115

The glass transition temperatures of the three HTMs were determined by differential scanning calorimetry (DSC, Figure 2d), measured during the second heating cycle. Substituting the hexyl group at the phenothiazine nitrogen in **SM1** with a phenyl-butoxy substituent in **SM2** resulted in an increase in *T*_g_ (109 °C vs 136 °C, respectively), as also seen in the literature.^53^ This is most likely a result of a more rigid system induced by the favorable π–π interactions of the newly added aromatic system, limiting molecular freedom. Oxidation of the sulfur atom (**SM3**) modifies the electronic configuration of the main phenothiazine scaffold, and as a result, further reduces the molecular freedom of the HTM, resulting in a higher *T*_g_ (147 °C with respect to **SM2**, its structural analog).

### Optoelectronic Properties

The semitransparency of the active layer in wide-band-gap (WBG) PSCs enables their potential application as bifacial photovoltaic devices. To fully exploit bifaciality, however, the HTM must exhibit negligible absorption in the visible range,^54^ thereby avoiding detrimental effects on photovoltaic efficiency by parasitic absorption-emission under rear-side illumination. To assess this property, the UV–vis absorption and photoluminescence spectra of **SM1**, **SM2**, and **SM3** were recorded. The normalized UV–vis absorption spectra are shown in Figure 2a, and the corresponding data are summarized in Table 1. The solution-state absorption features of the three HTMs are strongly dependent on their molecular structures. For **SM1**, the absorption band at 269 nm is assigned to a π–π* transition; this band undergoes a slight red shift to 278 nm upon replacement of the hexyl substituent with a phenyl-butoxy group in **SM2** and shifts further to 302 nm upon oxidation of the sulfur atom in **SM3**. The main absorption band, attributed to intramolecular charge transfer (ICT), appears at approximately 360, 370, and 367 nm for **SM1**, **SM2**, and **SM3**, respectively. The sulfoxide functionality in **SM3** induces a distinctive spectral response, characterized by (i) the absence of absorption beyond 400 nm, rendering the molecule more colorless, and (ii) a relatively narrow Stokes shift of 69 nm, compared to the broader shifts observed for **SM1** (149 nm) and **SM2** (142 nm), as illustrated in Figure 2a. Overall, the optical data confirm that all three HTMs exhibit minimal absorption within the visible spectral region, thereby demonstrating compatibility with the requirements of transparent and bifacial PSC architectures.

For efficient hole extraction from the perovskite (PSK) active layer, the HTM should possess a HOMO energy level slightly higher (by ∼0.2 eV) than the PSK valence band. Such alignment provides sufficient thermodynamic driving force for hole transfer, thereby maximizing the open-circuit voltage (*V*_OC_) and minimizing nonradiative losses. Accordingly, the HOMO level of an HTM must be carefully tuned with respect to the perovskite energy levels.

Although direct measurement is not easily experimentally accessible, the HOMO energy can be estimated from the oxidation potential of the electroactive species determined by cyclic voltammetry (CV).

The CV profiles of the three HTMs are shown in Figure 2b. For **SM1**, three distinct reversible oxidation peaks are observed. The first (lowest potential) and third (highest potential) processes correspond to oxidations localized on the phenothiazine core, consistent with those observed in the CV of the pristine scaffold,^55^ while the intermediate peak at ∼0.63 V is attributable to oxidation of the triphenylamine (TPA) units.^56^ The HOMO energy level was calculated using the relation *E*_HOMO_ [eV] = −(5.10 + *E*_1/2_ [*V* vs Fc^+^/Fc]), where *E*_1/2_ is the half-wave potential, defined as the average of the cathodic and anodic peak potentials. Based on this approach, the HOMO energy level of **SM1** is −5.38 eV, downshifted relative to that of Spiro-OMeTAD (−5.20 eV)^57^ and thus providing a more favorable alignment with the valence band of the WBG perovskite (−5.6 eV).^58^ Similar conclusions can be drawn for **SM2**. Substitution of the hexyl chain with a phenyl-butoxy group results in only a slight HOMO stabilization (−5.35 eV). In contrast, **SM3** displays a remarkably stabilized HOMO level (−5.63 eV), attributable to the electron-withdrawing sulfoxide functionality. However, this deep HOMO level results in unfavorable band alignment with the WBG perovskite employed in this study, suggesting that **SM3** would not be effective as an HTM under these conditions.

### Theoretical Analysis of Dyes

Figure 3 presents the highest occupied molecular orbitals (HOMOs) of the three small-molecule HTMs together with their corresponding oxidation potentials. The calculated values show qualitative agreement with the experimental CV data. Among the series, **SM3** is characterized by a markedly more stabilized frontier electronic structure, displaying a HOMO deeper than those of **SM1**and **SM2** by approximately 0.2 eV. A notable distinction lies in the orbital distribution: in **SM3**, the HOMO does not involve the sulfur atom, which is in a higher oxidation state due to its bond with oxygen. In contrast, the HOMOs of **SM1** and **SM2** display similar characteristics, with both showing significant sulfur orbital contributions. This difference indicates that oxidation of the sulfur atom substantially modifies the electronic structure of the PTZ core, reducing the contribution of sulfur orbitals to the π-system and stabilizing the HOMO. Accordingly, the redox behavior of **SM1** and **SM2** remains very similar, whereas **SM3** is clearly differentiated by the sulfoxide-induced stabilization of the frontier orbital.

**Figure 3:**
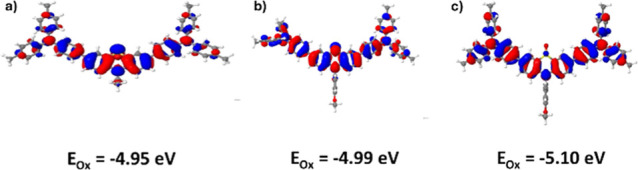
HOMOs of **SM1** (a), **SM2** (b), and **SM3** (c) and their respective oxidation potentials in the solvent.

### Electronic States

The presence of multiple reversible oxidation peaks in the CV profiles suggests the existence of several stable, energetically close oxidized states of the HTMs. On this basis, we tentatively attribute the additional anodic features to the population of low-lying electronic states of the oxidized HTMs. To test this hypothesis, the electronic structure of the oxidized species was examined by analyzing the transitions to the first and second excited doublet states. These states are found to be dominated by singly occupied molecular orbital (SOMO) transitions, namely, SOMO-1 → SOMO and SOMO-2 → SOMO transitions, respectively. Figure 4 depicts the SOMO, SOMO-1, and SOMO-2 of the investigated molecules. In **SM1** and **SM2**, the SOMO is primarily delocalized over the PTZ unit, whereas in **SM3**, it exhibits pronounced delocalization across the TPA units. SOMO-1 is largely localized on the TPA moieties, while SOMO-2 displays delocalization expanding onto both the TPA groups and the PTZ core. Table 2 summarizes the energy gaps associated with the D_0_ → D_1_ and D_0_ → D_2_ transitions. Although the calculated transition energies reproduce the experimental peaks only qualitatively, they nonetheless provide valuable insight, enabling assignment of the reversible features observed in the CV diagram to the formation of cationic species in their excited states. Notably, the D_0_ → D_1_ excitation of **SM3** is predicted to have an exceptionally low transition energy of ∼0.15 eV. This arises from the high degree of similarity between the electronic structures of the SOMO and SOMO-1 and accounts for the presence of only two peaks in the CV measurements, as opposed to the three observed for **SM1** and **SM2**. It is therefore likely that the low-lying D_1_ state is obscured beneath the band corresponding to the ground-state oxidation potential. It is therefore plausible that the low-lying D_1_ state does not generate a separate voltametric peak. Indeed, the calculated D_0_ → D_1_ gap of ∼0.15 eV implies a potential separation of only ∼0.15 V from the ground-state oxidation. Given the finite width of reversible CV waves, together with solvent/reorganization broadening and the qualitative nature of the calculated energies, these two oxidation processes would be expected to substantially overlap, making the D_1_-related feature difficult to resolve experimentally.

**Figure 4:**
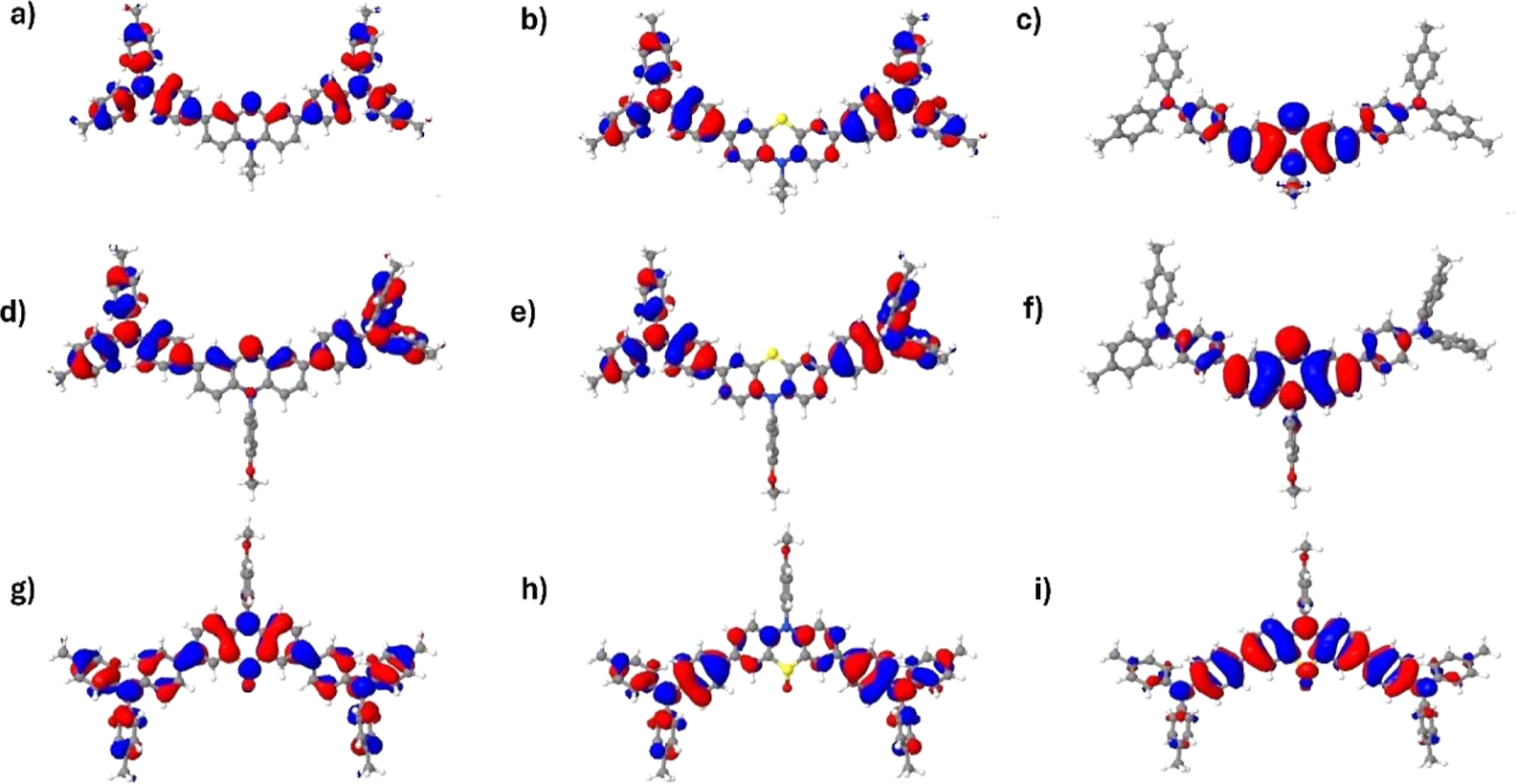
(a–c) Molecular orbitals involved in the first two electronic transitions of **SM1** (a) SOMO-2, (b) SOMO-1, and (c) SOMO. (d–f) Molecular orbitals involved in the first two electronic transitions of **SM2** (d) SOMO-2, (e) SOMO-1, and (f) SOMO. (g–i) Molecular orbitals involved in the first two electronic transitions of **SM3** (g) SOMO-2, (h) SOMO-1, and (i) SOMO.

**Table 2:** Transition Energies in eV and Electronic Transitions for the Scaffolds **SM1**, **SM2**, and **SM3**

scaffolds	D0 → D1	D0 → D2
SM1–2TPA	1.21	1.38
SM2–2TPA	1.22	1.37
SM3–2TPA	0.15	0.97

### Substituent Effects

Finally, the influence of TPA substituents on the redox properties of the three HTMs was examined. Figure 5 illustrates the impact of triphenylamine (TPA) substitution on the oxidation potentials of **SM1**, **SM2**, and **SM3**. The line graph reveals that the incorporation of two TPA units on all small molecules shifts the calculated oxidation potentials to less negative values.

**Figure 5:**
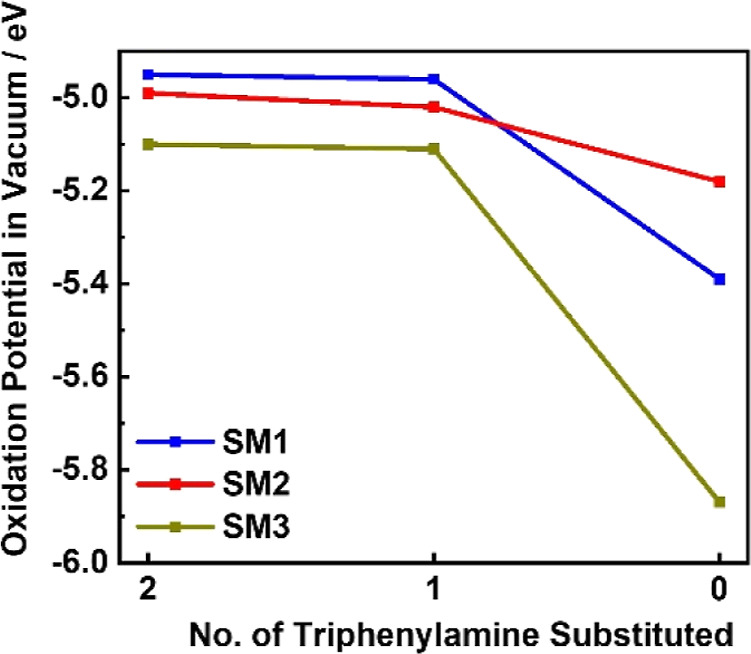
Effect of the TPAs on oxidation redox potential in vacuum (eV) for the scaffolds **SM1**, **SM2**, and **SM3**. In blue, the scaffold with two TPA substituents, in red, the scaffold with one TPA, and in brown, the scaffold with no TPA.

This trend demonstrates that molecules with a greater number of TPA substituents are more readily oxidized, consistent with the enhanced delocalization of the HOMO induced by conjugation with the TPA units. The numerical values reported in Table S1—see Supporting Information—further substantiate this correlation.

### Photovoltaic Performance of Small Molecules as HTMs in PSC Devices

To benchmark the performance of the newly developed small-molecule HTMs, the polymeric HTM poly­(triarylamine) PTAA was selected as the reference material, given its status as a state-of-the-art HTM.^9–11^ Indeed, a fairer comparison would be ensured by using Spiro-OMetaD, one of the most widely used small-molecule HTMs. However, as the present study focuses on semitransparent device architectures incorporating a sputtered ITO back electrode, Spiro-OMeTAD was not employed reference justified by its the poor compatibility with sputtered ITO,^59^ which leads to a pronounced reduction in device performance, manifested by a significant drop in the fill factor (FF), the appearance of an S-shaped *J*–*V* curve, and an overall decrease in power conversion efficiency (PCE), as illustrated in Figure S14. The device architecture consists of a TiO_2_ electron transport layer (ETL) combined with a double layer of SnO_2_ nanoparticles, followed by an ∼200 nm perovskite layer, an ISO/NEO passivation layer, and the HTMs (PTAA, **SM1**, and **SM2**), capped with a sputtered ITO electrode.

Although an accurate thickness determination of polymeric and small-molecule HTMs remains challenging, cross-sectional SEM images of the complete devices (Figure S15A–C) indicate comparable layer thicknesses among all the investigated HTMs. This observation is consistent with the nearly identical optical transmittance measured for films deposited on FTO substrates (Figure S15D), suggesting that the differences in device performance are not related to variations in HTM thickness or parasitic absorption. Figure 6 compares the photovoltaic parameters of the best-performing devices employing PTAA, **SM2**, and **SM1** as HTMs, with the corresponding values summarized in Table 3. **SM3** was excluded from this analysis, as no operational devices could be fabricated using this material. This outcome is consistent with the electrochemical data and computational simulation, due to the excessively deep HOMO energy level of **SM3** (−5.60 eV, *vide supra*), which prevents efficient hole extraction from the perovskite active layer. Despite this limitation in facilitating hole extraction for FAPbBr_3_-based perovskite absorbers, **SM3** may instead prove valuable in alternative perovskite systems featuring lower-lying valence bands, where better energetic alignment can be achieved. This highlights the potential for tuning the phenothiazine molecular framework to suit different absorber formulations, including emerging Pb-free perovskite materials.^60^ Both **SM1** and **SM2** deliver photovoltaic performances fully comparable to the reference PTAA under 1 sun illumination, despite the dopant formulation being optimized exclusively for the polymeric HTM.^61^ A more specific optimization of dopants for PTZ-based small molecules is out of the scope of the present paper, and it will be tackled in a forthcoming work.

**Figure 6:**
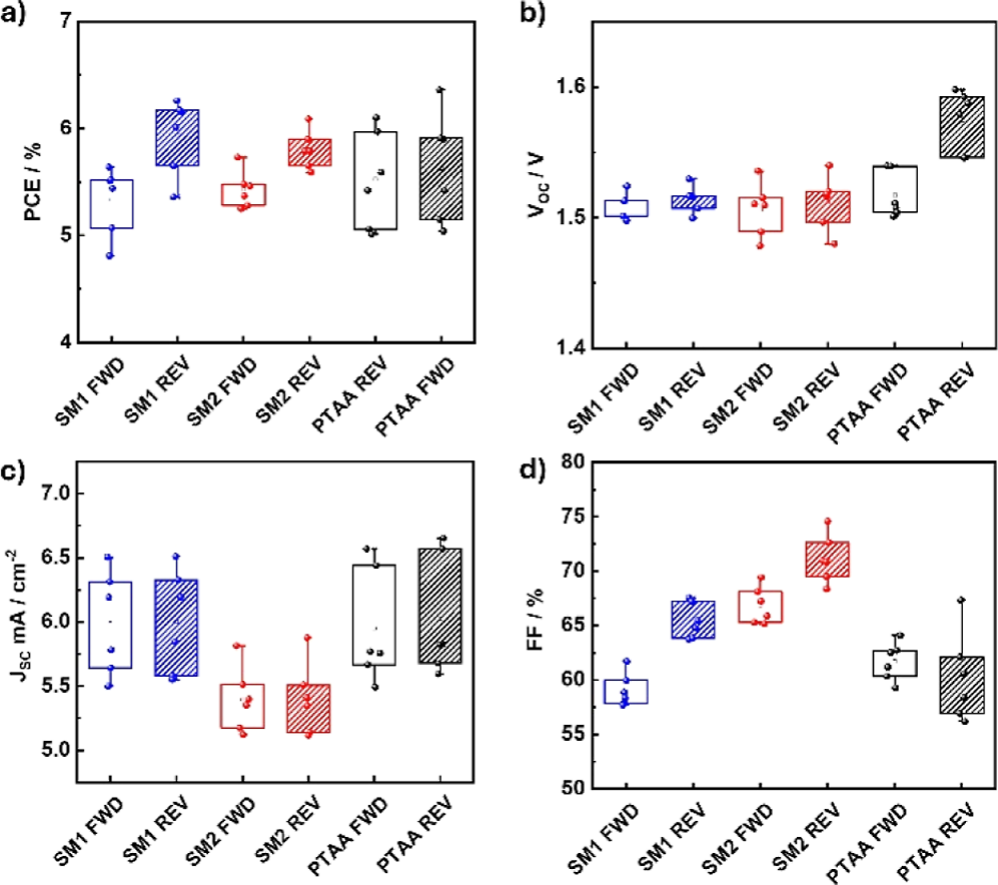
Statistical comparison of the photovoltaic performance of six semitransparent FAPbBr_3_ PSCs employing PTAA, **SM1**, and **SM2** as HTMs. (a) PCE (b), *V*_OC_, (c) current density, and (d) FF box plots.

**Table 3:** Statistical Comparison of the Photovoltaic Performance of Six Semi-transparent FAPbBr_3_ PSCs Employing PTAA, **SM1**, and **SM2** as HTMs Extracted from Forward and Reverse *J*–*V* Scans under 1 sun Illumination Reported in Figure 6

HTM	scan	*V*_OC_ (V)	FF (%)	*J*_SC_ (mA/cm^2^)	PCE (%)
**PTAA**	FWD	1.52 ± 0.02	60.2 ± 4.11	5.94 ± 0.44	5.52 ± 0.45
	REV	1.57 ± 0.02	61.7 ± 1.75	6.02 ± 0.46	5.63 ± 0.51
**SM2**	FWD	1.51 ± 0.02	66.8 ± 1.70	5.39 ± 0.25	5.42 ± 0.17
	REV	1.51 ± 0.02	71.2 ± 2.22	5.40 ± 0.28	5.80 ± 0.18
**SM1**	FWD	1.51 ± 0.01	59.1 ± 1.52	5.98 ± 0.40	5.33 ± 0.32
	REV	1.51 ± 0.01	65.4 ± 1.64	6.00 ± 0.40	5.93 ± 0.35

In forward scan, the average PCE values cluster around 5.3–5.9% for all HTMs, while in reverse scan, both small molecules slightly outperform PTAA, reaching average efficiencies of 5.93% (**SM1**) and 5.80% (**SM2**), compared to 5.63% for PTAA. This behavior is further corroborated by the champion devices (Table 4), where **SM1** and **SM2** achieve reverse-scan PCEs of 6.26% and 6.09%, respectively, closely matching the PTAA benchmark (6.39%).

**Table 4:** PV Parameters of Champion Devices in Figure 7A at 1 sun and Figure 7B 1000 lux for the three HTMs, as Integrated *J*_SC_, AVT, and LUE Values for the Champion Devices at 1 sun Conditions

HTM		scan	*V*_OC_/*V*	*J*_sc_/mA cm^–2^	FF/%	PCE/%	AVT/%	LUE/%
**PTAA**	1 SUN	FWD	1.54	6.15	67.9	6.06	63.8	4.07
		REV	1.54	6.17	65.4	6.39		
	indoor	FWD	1.25	6.85 × 10^–3^	64.7	14.4		
		REV	1.26	6.88 × 10^–3^	67.6	15.3		
**SM2**	1 SUN	FWD	1.51	5.81	65.3	5.73	65.6	3.99
		REV	1.51	5.87	68.3	6.09		
	indoor	FWD	1.35	6.34 × 10^–3^	61.8	15.7		
		REV	1.37	5.90 × 10^–3^	73.3	17.5		
**SM1**	1 SUN	FWD	1.50	6.50	57.7	5.64	64.7	4.05
		REV	1.50	6.51	63.8	6.26		
	indoor	FWD	1.30	6.39 × 10^–3^	66.5	16.4		
		REV	1.29	6.00 × 10^–3^	80.2	18.3		

A closer inspection of the photovoltaic parameters reveals that the three HTMs provide similar short-circuit current densities, with *J*_SC_ values in the 5.8–6.5 mA cm^–2^ range, indicating comparable optical absorption and charge collection efficiency. While PTAA exhibits a marginally higher *V*_OC_ (≈ 1.57 V in average) relative to **SM1** and **SM2** (≈ 1.50–1.51 V in average), the open-circuit voltage remains consistently above 1.5 V for all devices, suggesting that the perovskite absorber energetics and interfacial alignment are only weakly affected by the nature of the HTM. In contrast, the use of small-molecule HTMs has a pronounced impact on the fill factor. Both **SM1** and **SM2** display systematically higher FF values than PTAA on average, particularly in reverse scan, reaching up to 65.4% for **SM1** and 71.2% for **SM2**, compared to 61.7% for PTAA. The improvement in the FF is consistent with the transient characterization discussed below (Figures S17–S19), which highlights the role of interfacial charge extraction and transport dynamics. This enhancement may arise from improved molecular packing within the small-molecule layers, as well as the synergistic interaction of S, N, and O heteroatoms in the substituted phenothiazine molecules.^62,63^ These interactions can promote better contact with the perovskite and ITO layer. Despite this improvement, the overall FF values remain moderate, which can be primarily attributed to the use of sputtered ITO as the top electrode in semitransparent architectures. This configuration is known to introduce additional series resistance and potential interfacial damage during deposition, thereby limiting charge extraction efficiency, as widely reported in the literature.^59,64^

In Figure 7A,B, the *J*–*V* curves of the champion devices for each HTM are reported under 1 sun illumination and indoor conditions (1000 l×). In Figure S16A—see Supporting Information—to verify the accuracy of the current measurement, we report the external quantum efficiency (EQE) and the corresponding integrated *J*_SC_ for the champion devices of each HTM, while in Figure S16B, transmittance spectra are reported. The champion devices measured under 1 sun illumination further confirm the competitiveness of the phenothiazine-based small-molecule HTMs with respect to PTAA. The PTAA-based device delivers a *V*_OC_ of 1.54 V, a *J*_SC_ of 6.17 mA cm^–2^, and a FF of 65.4%, resulting in a reverse-scan PCE of 6.39%. Comparable performances are obtained with **SM1** and **SM2**. In particular, the **SM1** champion device reaches a *V*_OC_ of 1.50 V, a *J*_SC_ of 6.51 mA cm^–2^, and an FF of 63.8%, yielding a PCE of 6.26%, while **SM2** achieves a *V*_OC_ of 1.51 V, a *J*_SC_ of 5.87 mA cm^–2^, and a notably higher FF of 68.4%, corresponding to a PCE of 6.09%. Despite the slightly lower *V*_OC_ values for **SM1** and **SM2** compared to PTAA, observed also in the average values (Table 3), their higher FFs—and, in the case of **SM1**, enhanced current density—effectively compensate for this difference, leading to overall efficiencies that are fully comparable to the polymeric reference. Remarkably, these results are achieved without any specific dopant optimization for the small-molecule HTMs, highlighting their intrinsic suitability for efficient hole extraction and transport in wide-band-gap perovskite solar cells. To further validate the photovoltaic performance, maximum power point tracking (MPPT) measurements were performed **under 1 SUN and indoor conditions on the champion devices,** confirming a stable power output over time with negligible deviation from the PCE values derived from *J*–*V* measurements (Figure S16C,D).

**Figure 7:**
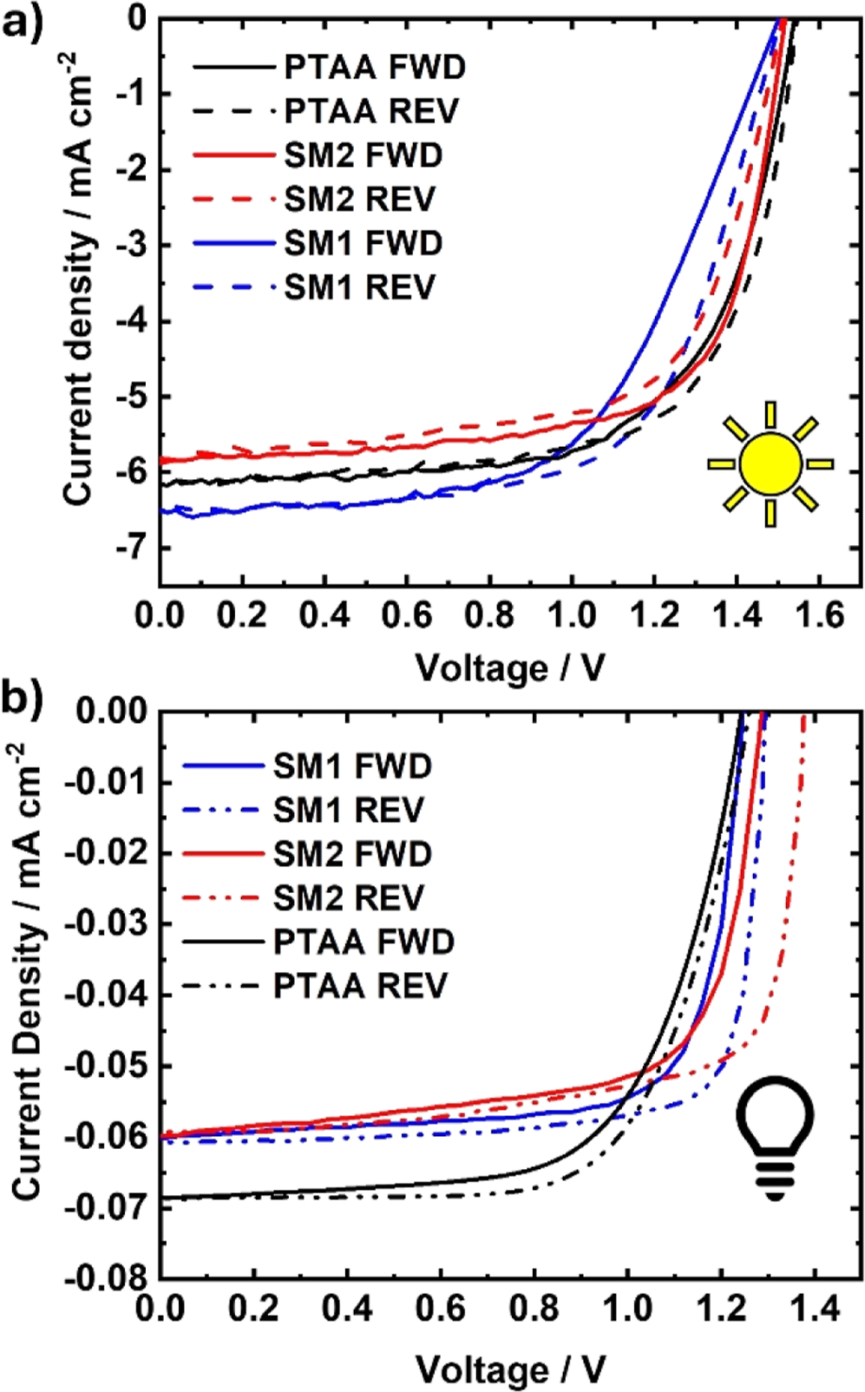
(a) *J*–*V* curves of the champion devices for each HTM in forward and reverse scan under 1 sun condition; (b) *J*–*V* curves of the champion devices for each HTM in forward and reverse scan under indoor conditions.

The indoor photovoltaic measurements (1000 lux) reveal some relevant differences in device operation between PTAA and the SM HTM, with the most significant distinction emerging in terms of *V*_OC_. Under indoor illumination, PTAA-based devices exhibit a *V*_OC_ of approximately 1.26 V, a value that is fully consistent with previously reported WBG perovskite devices employing PTAA as HTM under low-light conditions in the literature.^9^ In contrast, devices employing **SM1** and **SM2** show systematically higher *V*_OC_ values under indoor illumination, reaching up to ∼1.35 V. The higher *V*_OC_ under indoor illumination points to differences in interfacial energetics and charge extraction that become increasingly important under low carrier-density conditions. This *V*_OC_ enhancement represents a key advantage of the small-molecule HTMs, suggesting reduced nonradiative recombination losses and a more favorable energetic alignment at the perovskite/HTM interface, which becomes particularly critical under low carrier-density conditions typical of indoor lighting. The hysteresis index (HI), calculated as HI = (PCE_REV_ – PCE_FWD_)/PCE_REV_, yields values of ∼6% for PTAA and ∼10% for **SM1** and **SM2** under indoor conditions, indicating only moderate hysteresis. Despite this slightly higher HI for the SM-based devices, the stable steady-state output observed in MPPT measurements confirms that ion migration effects do not significantly impact the extracted performance metrics.

To further investigate the origin of the different current-collection behavior under indoor illumination, dark *J*–*V* measurements were analyzed to estimate the shunt (R_sh_) and series (R_s_) resistances of the devices (Figure S17 and Table S2). The extracted R_sh_ values are 53.2, 100.0, and 72.9 kΩ·cm^2^ for PTAA, **SM1**, and **SM2**, respectively, indicating that the small-molecule-based devices do not suffer from enhanced leakage currents compared to the **PTAA** reference. Therefore, the lower indoor *J*_SC_ observed for **SM1** and **SM2** cannot be directly attributed to shunt-related losses. On the other hand, the extracted *R*_s_ values increase from 208.4 Ω·cm^2^ for PTAA to 667.0 and 635.6 Ω·cm^2^ for **SM1** and **SM2**, respectively, suggesting that transport resistance may partially contribute to the different current-collection behavior under low-light conditions. However, considering the higher indoor *V*_OC_ exhibited by **SM1** and **SM2**, resistive effects alone cannot explain the observed performance trends, indicating that recombination and charge-extraction dynamics must also be taken into account.

To further elucidate the origin of the enhanced *V*_OC_ under indoor illumination, transient photovoltage (TPV) and transient photocurrent (TPC) measurements were performed (Figures S18–S20, Supporting Information). TPV measurements under high-injection conditions (1 sun bias) show slightly longer recombination lifetimes for PTAA-based devices compared to **SM1** and **SM2**, consistent with the marginally higher *V*_OC_ observed for PTAA under 1 sun. This indicates that recombination dynamics are not improved in SM-based devices under high carrier density. In contrast, intensity-dependent TPC measurements reveal a markedly different behavior at low illumination intensities. Under conditions comparable to indoor lighting, **SM1**- and **SM2**-based devices exhibit fast photocurrent rise and near-exponential decay, indicative of efficient and barrier-free hole extraction with negligible interfacial charge accumulation. This behavior supports reduced trap-assisted recombination and is consistent with the enhanced *V*_OC_ observed under low-light conditions. At higher illumination intensities, a slower decay component emerges, suggesting the onset of carrier accumulation at the HTM interface. These results indicate that while recombination dominates under high injection (TPV regime), efficient interfacial extraction and reduced trapping govern device operation at low carrier densities, enabling the higher *V*_OC_ observed for SM-based devices under indoor illumination. Light-intensity-dependent *V*_OC_ measurements yielded ideality factors of 3.05, 2.68, and 2.48 for PTAA, **SM1**, and **SM2**, respectively (Figure S21). The large ideality factors indicate that trap-assisted recombination contributes to the operation of all devices. Nevertheless, the slightly lower ideality factors of **SM1** and **SM2** suggest moderately reduced recombination losses compared to PTAA. Combined with the dark J–V analysis and the low-light TPC response, these results indicate that the enhanced indoor *V*_OC_ of the small-molecule HTMs originates from a combination of reduced trap-assisted recombination and favorable interfacial charge-extraction processes, rather than from differences in leakage currents.

In Figure S16B, Supporting Information, the transmittance graphs for the 3 devices with the three different HTMs are compared. Choosing different HTM values does not affect the overall device transmittance significantly. Similar AVT values—63.8%, 65.6%, and 64.7%, respectively, for PTAA, **SM1**, and **SM2**—are obtained in the visible range (380–780 nm) (Table 4). Consistently, the Light Utilization Efficiency (LUE, with LUE PCE*AVT) values reported in Table 4 are also comparable among the three HTMs, with values of 4.07 for PTAA, 3.99 for **SM2**, and 4.05 for **SM1**. This similarity reflects the nearly identical balance between PCE and AVT, confirming that the adoption of small-molecule HTMs preserves overall light management and device transparency while maintaining competitive photovoltaic performance.

To exploit the semitransparent feature of the device structure and HTMs, Figures S16A and S22, Supporting Information, report the EQE and JV characteristics under 1 sun illumination from both the front and rear sides. Further insights are provided by the bifacial characterization (Table S3). The bifaciality factor (BF) values are very similar for all investigated HTMs, indicating a comparable bifacial response of the devices. The PTAA-based device exhibits a BF of 0.81, while slightly higher values are obtained for the SM HTMs, namely, 0.82 for **SM2** and 0.84 for **SM1**. Although these differences are within a narrow range, they suggest that the use of phenothiazine-based SM does not penalize rear-side operation. Overall, the data confirm that PTAA, **SM1**, and **SM2** are equally suitable for semitransparent and bifacial perovskite solar cell architectures. An initial assessment of device stability was performed for **SM1**- and **SM2**-based devices, while PTAA-based devices have already been extensively investigated in the literature.^10^ Representative devices employing **SM1** and **SM2** were subjected to ISOS-D1 and ISOS-D2 protocols. The ISOS-D1 test consists of storing the devices under ambient environmental conditions, whereas the ISOS-D2 test involves storage in the dark at 85 °C. The devices exhibited stable performance over more than 1200 h under ISOS-D1 conditions, with no significant loss in efficiency. Under ISOS-D2 conditions, only a slight decrease in performance was observed after 1000 h, demonstrating the robustness of the small-molecule HTMs (Figure S23), comparable to that reported for PTAA.

## Conclusions

In this work, three novel small-molecule (**SM1–SM3**) based on a phenothiazine scaffold were designed, synthesized, and evaluated for application as hole-transporting materials in wide-band-gap PSCs. Guided by molecular engineering strategies, structural modifications were introduced through substitution at the PTZ nitrogen and oxidation of the PTZ sulfur, enabling systematic tuning of the electronic and optical properties. Computational and electrochemical analyses revealed that both **SM1** and **SM2** exhibit favorable HOMO energy levels, closely aligned with the valence band of WBG perovskites, thereby supporting efficient hole extraction. In fact, this translated to comparable device performances with respect to those of the reference PTAA. More in detail, the slightly lower *V*_OC_ (still exceeding 1.5 V, though) is counterbalanced by a higher FF and, in the case of **SM1**, also by a better current density (even higher than 6 mA cm^–2^). The latter results are quite remarkable considering that the dopant nature and concentration have been optimized for PTAA only. These results are tentatively ascribed to a better filming of the small molecules compared with the polymeric HTM. When tested under simulated indoor lighting (1000 lux), both **SM1** and **SM2** clearly outperformed the polymeric HTM, thanks to a consistently higher *V*_OC_ (**SM1**) or FF (**SM2**), indicating moderately reduced trap-assisted recombination and more favorable charge extraction dynamics at low light intensities. This characteristic makes the SM-based HTMs especially attractive for indoor and low-light photovoltaic applications. Overall, these results establish phenothiazine as a versatile and low-cost platform for the development of novel HTMs. **SM1** and **SM2**, in particular, emerge as promising candidates for transparent and bifacial PSCs, combining a suitable energy-level alignment, good thermal stability, and optical transparency. This work underscores the potential of rational PTZ-based molecular design in advancing the performance, stability, and commercial viability of next-generation perovskite photovoltaics across both outdoor and indoor operating conditions.

## Experimental Section

### Materials

Phenothiazine, sodium hydride (60% dispersion in mineral oil), iodohexane, RuPhos-Pd-G2, 3-chloroperbenzoic acid, *p*-bromotoluene, dichlorobis­(triphenylphosphine)­palladium­(II), tetrakis­(triphenylphosphine)­palladium(0), and sodium diethyldithiocarbamate trihydrate were all purchased from Sigma-Aldrich and used as received. 1-Bromo-4-butoxybenzene was purchased from Maybridge Chemicals and used as received. *p*-Toluidine was purchased from Carlo-Erba and used as received. Bis­(pinacolato)­diboron was purchased from TCI Chemicals and used as received. *N*-Bromosuccinimide and Aliquat 336 were purchased from ABCR and used as received. Solvents were all ACS reagent grade, purchased from Sigma-Aldrich and used as received, without further purification.

For the device’s fabrication. The chemicals used throughout this work comprised anhydrous dimethyl sulfoxide (DMSO, 99.9%) and anhydrous ethyl acetate (99%), together with titanium diisopropoxide bis­(acetylacetonate), lithium bis­(trifluoromethanesulfonyl)­imide (Li-TFSI, 99.95%), 4-*tert*-butylpyridine (tBP, 96%), and niobium ethoxide, all supplied by Sigma-Aldrich. High-purity lead bromide (PbBr_2_, 99.99%) was obtained from TCI Chemicals. Formamidinium bromide (FABr, 99.98%), neo-pentylammonium chloride, and iso-pentylammonium chloride were purchased from Great Cell Materials. Poly­(triarylamine) (PTAA, Mw ≈ 10 kDa) was sourced from Solaris Chem. A 15 wt % aqueous colloidal solution of tin­(IV) oxide was acquired from Alfa Aesar. Additionally, an Al_2_O_3_ nanoparticle dispersion (particle size <50 nm, 20 wt % in isopropanol) and the ionic liquid BMIMBF_4_ were obtained from Sigma-Aldrich.

The synthetic methodology for PTZ scaffolds is shown in Scheme S1.

### Synthetic Procedures

#### Synthesis of **i**

Into a three-necked round-bottom flask (50 mL), phenothiazine (0.199 g, 1 mmol, 1 equiv) was dissolved in dry DMF (20 mL) and NaH (0.048 g, 2 mmol, 2 equiv) was added at 0 °C and stirred for 1 h. 1-Iodohexane (0.318 g, 0.221 mL, 1.5 mmol, 1.5 equiv) was then slowly added dropwise, over 15 min. The reaction was then left stirring for 4 h at room temperature and was checked for its completion by TLC. Once all phenothiazine had been consumed, the reaction mixture was quenched with water and extracted with (3 × 10 mL) H_2_O, brine (3 × 10 mL), and finally with ethyl acetate (3 × 10 mL). The crude product was purified by column chromatography with petroleum ether, yielding a white solid. η­(**i**) – 78% (0.221 g).

^1^H NMR (600 MHz, DMSO-*d*_6_) δ: 7.19 (ddd, *J* = 8.6, 7.3, 1.5 Hz, 2H), 7.14 (dd, *J* = 7.6, 1.5 Hz, 2H), 7.01 (dd, *J* = 8.2, 1.2 Hz, 2H), 6.93 (td, *J* = 7.4, 1.2 Hz, 2H), 3.85 (t, *J* = 7.0 Hz, 2H), 1.71–1.62 (m, 2H), 1.40–1.33 (m, 2H), 1.23 (h, *J* = 4.0 Hz, 4H), 0.85–0.78 (m, 3H). ^13^C NMR (151 MHz, DMSO-*d*_6_) δ: 144.82, 127.59, 127.12, 123.62, 122.42, 115.84, 46.40, 30.82, 26.20, 25.83, 22.06, 13.83.

#### Synthesis of **ii**

To a solution of compound **i** (0.221 g, 0.78 mmol, 1 equiv) in dichloromethane (20 mL) in a three-necked round-bottom flask (100 mL), a solution of Br_2_ (0.623 g, 0.201 mL, 3.9 mmol, 5 equiv) in dichloromethane (10 mL) was added dropwise over 15 min. After 5–10 min following the completion of bromine addition, a TLC control (Silica TLC plates, eluent: dichloromethane) was performed to assess the completion of the reaction. The reaction was then quenched with Na_2_SO_3_ and water until a color change had been observed from purple to white. At that point, the final product was extracted with dichloromethane (3 × 10 mL), and the organic layer was dried over Na_2_SO_4_. The solvent was subsequently removed under reduced pressure, yielding a light-yellow oil. η­(**ii**) – 95% (0.3279 g).

^1^H NMR (600 MHz, CDCl3) δ: 7.24–7.20 (m, 4H), 6.67 (d, *J* = 8.6 Hz, 2H), 3.74 (s, 2H), 1.78–1.67 (m, 2H), 1.42–1.34 (m, 2H), 1.32–1.22 (m, 4H), 0.89–0.82 (m, 3H). ^13^C NMR (151 MHz, CDCl_3_) δ: 144.32, 130.26, 129.87, 126.61, 118.85, 116.80, 114.90, 47.76, 31.51, 26.76, 26.64, 22.70, 14.11.

#### Synthesis of **iii**

A 5 mL microwave (MW) vial reactor was left overnight in an anhydrous oven. After the reactor was allowed to cool down to room temperature and was thoroughly degassed with nitrogen. Na^t^BuO (0.115 g, 1.20 mmol, 1.2 equiv) was inserted into the vial, and 3 vacuum argon cycles were conducted with the help of a heat gun. Phenothiazine (0.199 g, 1.00 mmol, 1 equiv), 1-bromo-4-butoxybenzene (0.252 g, 1.10 mmol, 1.1 equiv), and chloro­(2-dicyclohexylphosphino-2′,6′-diisopropoxy-1,1′-biphenyl)­[2-(2′-amino-1,1′-biphenyl)]­palladium­(II) (RuPhos-Pd-G2) (0.015 g, 0.02 mmol, 0.02 equiv) were all introduced at once, closed with an aluminum crimp seal, and thoroughly flushed with nitrogen for at least 15 min. Anhydrous toluene (2 mL) was added to the reaction vial and was left to react overnight at 110 °C. The reaction was quenched with water and extracted with dichloromethane (3 × 5 mL). The organic fraction was further washed with brine (3 × 5 mL) and finally dried over anhydrous Na_2_SO_4_. The crude was purified via column chromatography (petroleum ether: dichloromethane), yielding a white solid. η­(**iii**) – 87% (303 mg).

^1^H NMR (600 MHz, CDCl_3_) δ: 7.29 (d, *J* = 6.7 Hz, 2H), 7.12–7.07 (m, 2H), 6.99 (dd, *J* = 7.5, 1.7 Hz, 2H), 6.85–6.75 (m, 4H), 6.20 (dd, *J* = 8.2, 1.3 Hz, 2H), 4.04 (t, *J* = 6.5 Hz, 2H), 1.87–1.79 (m, 2H), 1.54 (dt, *J* = 14.8, 7.5 Hz, 2H), 1.02 (t, *J* = 7.4 Hz, 3H) ^13^C NMR (151 MHz, CDCl_3_) δ: 158.99, 144.83, 133.16, 132.34, 126.94, 126.73, 122.34, 119.74, 116.48, 115.77, 68.12, 31.47, 19.43, 14.02.

#### Synthesis of **iv**

To a solution of compound **iii** (0.271 g, 0.78 mmol, 1 equiv) in dichloromethane (5 mL) in a three-necked round-bottom flask (25 mL), a solution of Br_2_ (0.599 g, 0.193 mL, 3.75 mmol, 5 equiv) in dichloromethane (1 mL) was added dropwise over 15 min. After 5–10 min following the completion of bromine addition, a TLC control (petroleum ether: dichloromethane, 9:1) was performed to assess the completion of the reaction. The reaction was then quenched with Na_2_SO_3_ and water until a color change had been observed from purple to white. At that point, the final product was extracted with dichloromethane (3 × 50 mL), and the organic layer was dried over Na_2_SO_4_. The solvent was subsequently removed under reduced pressure, yielding a white solid. η­(**iv**) – 96% (366.2 mg).

^1^H NMR (600 MHz, CDCl_3_) δ: 7.24–7.19 (m, 2H), 7.11–7.07 (m, 2H), 7.06 (d, *J* = 2.3 Hz, 2H), 6.90 (dd, *J* = 8.8, 2.3 Hz, 2H), 6.01 (d, *J* = 8.8 Hz, 2H), 4.03 (t, *J* = 6.5 Hz, 2H), 1.86–1.79 (m, 2H), 1.56–1.49 (m, 2H), 1.01 (t, *J* = 7.4 Hz, 3H). ^13^C NMR (151 MHz, CDCl_3_) δ: 159.32, 143.65, 132.34, 131.89, 129.82, 128.84, 121.13, 117.06, 116.79, 114.64, 68.19, 31.43, 19.42, 14.01.

#### Synthesis of **v**

To a solution of **iv** (0.150 g, 0.297 mmol, 1 equiv) in dichloromethane (5 mL), 3-chloroperbenzoic acid (0.051 g, 0.297 mmol, 1 equiv) was slowly added at 0 °C. The reaction was completed in 5 h. The reaction was quenched with a saturated solution of Na_2_CO_3_, which was then extracted with dichloromethane (3 × 20 mL). The organic layer was dried over anhydrous Na_2_SO_4_. The crude was purified via column chromatography (petroleum ether: ethyl acetate), yielding a white solid. η­(**v**) – 88% (136.2 mg).

^1^H NMR (600 MHz, CDCl_3_) δ: 8.07 (d, *J* = 2.3 Hz, 2H), 7.47 (dd, *J* = 9.1, 2.3 Hz, 2H), 7.26–7.22 (m, 2H), 7.18–7.14 (m, 2H), 6.66 (d, *J* = 9.1 Hz, 2H), 4.07 (t, *J* = 6.5 Hz, 2H), 1.89–1.81 (m, 2H), 1.59–1.52 (m, 2H), 1.03 (t, *J* = 7.4 Hz, 3H). ^13^C NMR (151 MHz, CDCl_3_) δ: 160.12, 138.04, 135.66, 133.92, 131.06, 130.80, 123.81, 119.53, 117.12, 114.27, 68.35, 31.37, 19.40, 13.99.

#### Synthesis of **vi**

A 5 mL microwave (MW) vial reactor was left overnight in an anhydrous oven. Then, the reactor was allowed to cool down to room temperature and was thoroughly degassed with nitrogen. Na^t^BuO (0.115 g, 1.20 mmol, 1.2 equiv) was inserted into the vial, and 3 vacuum argon cycles were conducted with the help of a heat gun. *P*-Toluidine (0.107 g, 1.00 mmol, 1 equiv), *p*-bromotoluene (0.180 g, 1.05 mmol, 1.1 equiv), and chloro­(2-dicyclohexylphosphino-2′,6′-diisopropoxy-1,1′-biphenyl)­[2-(2′-amino-1,1′-biphenyl)]­palladium­(II) (RuPhos-Pd-G2) (0.015 g, 0.02 mmol, 0.02 equiv) were all introduced at once, closed with an aluminum crimp seal, and thoroughly flushed with nitrogen for at least 15 min. Anhydrous toluene (2 mL) was added to the reaction vial and was left to react overnight at 110 °C. The reaction was quenched with water and extracted with dichloromethane (3 × 5 mL). The organic fraction was further washed with brine (3 × 5 mL) and finally dried over anhydrous Na_2_SO_4_. The crude was purified via column chromatography (petroleum ether: dichloromethane), yielding a white solid. η­(**iii**) – 78% (154.5 mg).

^1^H NMR (600 MHz, CDCl_3_) δ: 7.08–7.05 (m, 4H), 6.97–6.93 (m, 4H), 2.29 (s, 6H). ^13^C NMR (151 MHz, CDCl_3_) δ: 141.28, 130.33, 129.96, 118.06, 20.76.

#### Synthesis of **vii**

A 5 mL microwave (MW) vial reactor was left overnight in an anhydrous oven. Then, the reactor was allowed to cool down to room temperature and was thoroughly degassed with nitrogen. Na^t^BuO (0.058 g, 0.600 mmol, 1.2 equiv) was inserted into the vial, and 3 vacuum argon cycles were conducted with the help of a heat gun. Compound **vi** (0.099 g, 0.500 mmol, 1 equiv), bromobenzene (0.082 g, 0.055 mL, 0.525 mmol, 1.05 equiv), and chloro­(2-dicyclohexylphosphino-2′,6′-diisopropoxy-1,1′-biphenyl)­[2-(2′-amino-1,1′-biphenyl)]­palladium­(II) (RuPhos-Pd-G2) (0.015 g, 0.02 mmol, 0.02 equiv) were all introduced at once, closed with an aluminum crimp seal, and thoroughly flushed with nitrogen for at least 15 min. Anhydrous toluene (2 mL) was added to the reaction vial and was left to react overnight at 110 °C. The reaction was quenched with water and extracted with dichloromethane (3 × 5 mL). The organic fraction was further washed with brine (3 × 5 mL) and finally dried over anhydrous Na_2_SO_4_. The crude was purified via column chromatography (petroleum ether), yielding a white solid. η­(**vii**) – 53% (72.5 mg).

^1^H NMR (600 MHz, CDCl_3_) δ: 7.22–7.18 (m, 2H), 7.07–7.01 (m, 6H), 7.00–6.97 (m, 4H), 6.93 (tt, *J* = 7.4, 1.1 Hz, 1H), 2.31 (s, 6H). ^13^C NMR (151 MHz, CDCl_3_) δ δ: 148.41, 145.60, 132.44, 129.97, 129.16, 124.59, 123.09, 121.83, 20.93.

#### Synthesis of **viii**

Compound **vii** (0.086 g, 0.313 mmol, 1 equiv) was dissolved in 25 mL of cold CHCl_3_ in a 25 mL round-bottom flask. *N*-Bromosuccinimide (0.071 g, 0.399 mmol, 1.05 equiv) was slowly added in small portions at 0 °C. The reaction was left to react for 4 h in the dark. Then, the reaction was quenched with water and extracted with dichloromethane (3 × 20 mL) and washed with deionized water (3 × 20 mL) and brine (3 × 20 mL). The organic fractions were collected and dried over anhydrous Na_2_SO_4_. The crude was purified by column chromatography (100% petroleum ether), yielding a white solid. η­(**vii**) – 99% (133.9 mg).

^1^H NMR (600 MHz, CDCl_3_) δ: 7.29–7.26 (m, 2H), 7.07 (d, *J* = 8.1 Hz, 4H), 6.98 (d, *J* = 8.4 Hz, 4H), 6.91–6.87 (m, 2H), 2.32 (s, 6H). ^13^C NMR (151 MHz, CDCl_3_) δ: 147.57, 145.07, 133.06, 132.05, 130.13, 124.81, 124.04, 113.74, 20.95.

#### Synthesis of **ix**

A round-bottom flask (50 mL), dried overnight in an anhydrous oven, was flushed thoroughly with nitrogen. Compound **viii** (0.539 g, 1.53 mmol, 1 equiv), bis­(pinacolato)­diboron (0.485 g, 1.91 mmol, 1.25 equiv), dichlorobis­(triphenylphosphine)­palladium­(II) (Pd­(PPh_3_)_2_Cl_2_) (0.032 g, 0.0450 mmol, 0.03 equiv), and KOAc (0.049 g, 0.5 mmol, 3 equiv) were all inserted at once, closed with an aluminum crimp seal, and thoroughly flushed with nitrogen for at least 15 min. Anhydrous 1,4-dioxane (10 mL) was finally added to the flask, and the mixture was left to react overnight at 100 °C. The reaction was quenched with a saturated solution of NaCl­(aq), and the organic components were extracted with dichloromethane (3 × 50 mL). The organic layer was dried over anhydrous Na_2_SO_4_, and the evaporated solvent was removed under reduced pressure. The crude product was purified by column chromatography using petroleum ether: ethyl acetate as the eluent (97:3), yielding a white solid. η­(**ix**) – 67% (406.5 mg).

^1^H NMR (600 MHz, CDCl_3_) δ: 7.65–7.59 (m, 2H), 7.08–7.05 (m, 4H), 7.02–6.99 (m, 4H), 6.98–6.94 (m, 2H), 2.32 (s, 6H), 1.32 (s, 12H). ^13^C NMR (151 MHz, CDCl_3_) δ: 151.07, 145.00, 135.88, 133.25, 130.08, 125.40, 120.64, 83.61, 24.98, 20.99.

#### General Synthesis of SM1–3

A 30 mL microwave (MW) vial reactor was left overnight in an anhydrous oven. Then, the reactor was allowed to cool down to room temperature and was thoroughly degassed with nitrogen. Anhydrous toluene, as well as a 2 M K_2_CO_3_(aq) solution, was also previously degassed with argon. Scaffolds **ii**, **iv**, and **v** (0.1 mmol, 1 equiv), compound **ix** (0.108 g, 0.27 mmol, 2.7 equiv), and tetrakis­(triphenylphosphine)­palladium(0) (Pd­(PPh_3_)_4_) (0.058 g, 0.05 mmol, 0.05 equiv) were all weighed and inserted into the reaction flask along with a few drops of Aliquat 336 and closed with an aluminum crimp seal. The solids were flushed with nitrogen before the addition of anhydrous toluene (5 mL) and 2 M K_2_CO_3_(aq) (6.3 equiv) solution. The reaction was left to react for 48 h at 110 °C. The cooled reaction mixture was then added dropwise into methanol (40 mL) and stirred overnight, resulting in the precipitation of the small molecule. The small molecules were purified from palladium contamination via metal scavenging using sodium diethyldithiocarbamate trihydrate (NaDTC). The procedure went as follows: the small molecule was dissolved in tetrahydrofuran at a concentration of 10 mg/mL. NaDTC (50 equiv), with respect to the quantity of residual palladium from the synthesis, was added to the solution and left mixing overnight at room temperature. The purified small molecule was recovered via precipitation in methanol:

η­(**SM1**) – 87% (72.1 mg) ^1^H NMR (600 MHz, THF-d8) δ: 7.43–7.39 (m, 4H), 7.38–7.34 (m, 4H), 7.07–6.93 (m, 22H), 3.93 (t, *J* = 7.1 Hz, 2H), 2.28 (s, 12H), 1.83 (p, *J* = 7.6 Hz, 2H), 1.54–1.43 (m, 2H), 1.34 (td, *J* = 7.0, 3.5 Hz, 4H), 0.93–0.84 (m, 3H). ^13^C NMR (151 MHz, THF-d8) δ: 148.34, 146.47, 144.89, 135.98, 134.31, 133.21, 130.66, 127.70, 125.86, 125.77, 125.41, 123.83, 116.42, 32.55, 27.83, 27.51, 23.59, 20.89, 14.40.

η­(**SM2**) – 86% (77.1 mg) ^1^H NMR (600 MHz, THF-d8) 7.37–7.32 (m, 6H), 7.23 (d, *J* = 2.2 Hz, 2H), 7.18 (d, *J* = 8.9 Hz, 2H), 7.06–7.03 (m, 10H), 7.00–6.92 (m, 12H), 6.21 (d, *J* = 8.6 Hz, 2H), 4.07 (t, *J* = 6.4 Hz, 2H), 2.28 (s, 12H), 1.82 (dt, *J* = 14.3, 6.4 Hz, 2H), 1.56 (dq, *J* = 14.8, 7.4 Hz, 2H), 1.02 (t, *J* = 7.4 Hz, 3H). ^13^C NMR (151 MHz, THF-d8) δ: 160.24, 148.33, 146.45, 144.20, 135.85, 134.10, 133.20, 133.02, 132.78, 130.65, 127.53, 125.46, 125.39, 125.00, 123.82, 120.74, 117.31, 116.75, 32.41, 20.89, 20.24, 14.26.

η­(**SM3**) – 42% (38.0 mg) ^1^H NMR (600 MHz, CDCl_3_) 8.16 (d, *J* = 2.2 Hz, 2H), 7.58 (dd, *J* = 8.9, 2.3 Hz, 2H), 7.45–7.41 (m, 4H), 7.33 (d, *J* = 8.9 Hz, 2H), 7.18 (d, *J* = 9.0 Hz, 2H), 7.11–7.00 (m, 20H), 6.81 (d, *J* = 8.9 Hz, 2H), 4.09 (t, *J* = 6.4 Hz, 2H), 2.32 (s, 12H), 1.90–1.83 (m, 2H), 1.57 (q, *J* = 7.4 Hz, 2H), 1.04 (t, *J* = 7.4 Hz, 3H). ^13^C NMR (151 MHz, CDCl_3_) δ: 147.88, 145.26, 137.97, 134.97, 132.92, 132.23, 131.46, 130.77, 130.10, 129.46, 127.30, 124.96, 122.71, 117.99, 116.87, 31.44, 20.98, 19.44, 14.03.

### Characterization

NMR spectra for ^1^H and ^13^C nuclei were recorded on a JEOL ECZ-R 600, working at 600 MHz. Thermogravimetric analysis (TGA) has been performed with a TGA TAQ 600 (TA Instruments) under a nitrogen atmosphere (100 mL/min) with a heating gradient of 10 °C/min from 60 to 800 °C. For each analysis, 5 mg of the sample was weighed in an alumina pan that was previously cleaned at 1000 °C with an oxidant flame. Differential scanning calorimetric (DSC) analysis was performed with a DSC TAQ 200 (TA Instruments) in a nitrogen atmosphere with a heating gradient of 30 °C/min, in the range from −50 to 250 °C under a nitrogen flow (50 mL/min) and after 10 min of equilibration at −50 °C. Around 10 mg of the sample was placed in an aluminum-capped pan, and an identical one was used as a reference. UV–vis absorption spectra were recorded in a dichloromethane solution in a quartz cuvette with an Agilent Cary 5000 Bio spectrophotometer and using pure solvent as a reference. Emission spectra have been acquired with a Fluorolog TCSPC Horiba Jobin Yvon Spectrofluorometer, with a xenon lamp and slits varying from 1 to 15 nm, using a dichloromethane solution with absorption = 0.1. Cyclic voltammetry analysis was performed with an SP-300 BIOLOGIC instrument, in a three-electrode setup (working electrode and counter electrode were a glassy carbon and platinum spiral, respectively) using dichloromethane as a solvent and tetrabutylammonium hexafluorophosphate (TBA^+^PF_6_^–^) as a supporting electrolyte. The scan rate was set to 50 mV/s, and the scan was performed from −1.2 to 1.5 V using AgCl/Ag as a reference electrode and Fc^+^/Fc as an internal reference. Fourier transform infrared spectroscopy was performed on an Invenio Spectrometer by Bruker, using Attenuated Total Reflectance (ATR) mode.

### Computational Details

Redox potentials were calculated using density functional theory (DFT) at the M062X/def2-TZVP level of theory in dichloromethane (CH_2_Cl_2_). The absorption spectra of **SM1**, **SM2**, and **SM3** are obtained with time-dependent density functional theory (TD-DFT) at the level of theory M062X/def2-TZVP in dichloromethane (CH_2_Cl_2_). The solvent effect was introduced into the calculation using the polarizable continuum model (PCM).

### Device Fabrication and Testing

FTO conductive glass substrates (7 Ω/sq) from Pilkington were laser-etched using a nanosecond UV laser to create the desired layout. This layout consists of four small cells, each with an active area of 0.3 cm^2^, on a 2.5 cm × 2.5 cm substrate. After laser processing, the substrates were cleaned to remove dust and organic residues generated during cutting. The cleaning procedure involved an initial ultrasonic bath in a soap-water solution for 10 min at 40 °C. This was followed by rinsing with deionized water in a separate 10 min ultrasonic bath at 40 °C. Finally, the substrates underwent a final ultrasonic bath using isopropanol. Clean substrates were annealed at 465 °C. A solution of 0.16 M Ti­(AcAc)_2_ and 0.4 M AcAc in EtOH was then deposited using the spray pyrolysis technique (air as gas carrier, 1.6 bar pressure, nozzle angle ≈45°, 10 cycles at 10 s intervals). Before perovskite deposition, the substrates with the TiO_2_ electron transport layer (ETL) underwent 30 min of UV-OZONE treatment to further clean them, improve their wettability, and facilitate perovskite deposition. In a N_2_ environment, substrates were annealed at 60 °C. 80 μL of the FAPbBr_3_ perovskite solution (prepared with 1 M PbBr_2_ from TCI and 1 M FaBr from Sigma-Aldrich in DMSO) was deposited using spin coating at 4000 rpm for 20 s. After 10 s from the spin’s start, 200 μL of ethyl acetate was added dropwise as an antisolvent to facilitate perovskite conversion. Finally, the substrates were moved to 80 °C to complete the conversion and remove excess solvent. Finally, triarylamine-modified phenothiazine small molecules (**SM1** and **SM2**) were tested as HTMs. PTAA (Sigma-Aldrich) and Spiro-OMeTAD were included as reference HTMs. PTAA (10 mg/mL) was diluted in toluene and doped with a solution of 5 μL/mL Li-salt (170 mg/mL LiTFSI in acetonitrile) and 10 μL/mL of 4-*tert*-butylpyridine; it was then spin-coated onto cooled substrates at 2000 rpm for 40 s. **SM1** and **SM2** were deposited following the same processing conditions as PTAA. As an alternative to PTAA and small molecules, spiro-OMeTAD was deposited by spinning 90 μL of the spiro-OMeTAD solution in CB (73.5 mg/mL) doped with tBP (26.8 μL/mL), LiTFSI (16.6 μL/mL) (from the stock solution in CAN (520 mg/mL) and FK209 Co­(III) TFSI (7.2 μL/mL)), at 4000 rpm for 1 min, with an acceleration of 1000 rpm/s. ITO as a back electrode was sputtered onto the samples using an industrial magnetron sputtering system (KENOSISTEC S.R.L., KS 400 In-Line). The sputtering process occurred at a pressure of 1.1 × 10^–3^ mbar with 90 W of RF power. Inert argon gas was purged into the chamber at a flow rate of 40 sccm. The samples were moved past the ITO target at a speed of 120 cm/min for 200 cycles using a sample holder. This process resulted in a 200 nm thick ITO electrode with a sheet resistance of 25 Ω/sq, as measured by a four-probe unit within the Arkeo Platform (Cicci Research S.r.L.).

Devices were characterized under simulated sunlight using a Class A solar simulator (ABET Sun 2000) calibrated to AM 1.5 conditions and a 100 mW/cm^2^ illumination intensity. A certified reference silicon cell (RERA Solutions RR-1002) was used for calibration. Current density–voltage (*J*–*V*) curves were measured in both forward and reverse bias directions with a scan rate of 300 mV/s and a voltage step of 50 mV. A commercial 4-wire source meter with multiple channels (Arkeo-Ariadne, Cicci Research s.r.l.) was employed for these measurements. Indoor measurements were carried out using the same experimental setup and software, with a 6500 K lamp as the light source. For External Quantum Efficiency (EQE) measurements, Transient Photovoltaic (TPV) and Transient Photocurrent (TPC), a commercial setup (Arkeo-Ariadne, Cicci Research s.r.l.) equipped with a 300 W xenon lamp was used. For EQE, this setup enables spectral acquisition from 300 to 1100 nm with a resolution of 2 nm. The Average Visible Transmittance (AVT) values were calculated using a method taken from the literature.^58^ The Light Utilization Efficiency (LUE) value was calculated through the following formula: LUE = PCE*AVT. Scanning Electron Microscopy (SEM) measurements were performed using a high-resolution SEM device with an FEG Schottky electron source (TESCAN MIRA). Dark current density–voltage (dark *J*–*V*) measurements were performed using a commercial setup (Arkeo-Ariadne, Cicci Research s.r.l.). The measurements were carried out in the absence of illumination over the voltage range required for the analysis of the low-bias and high-forward-bias regions. The shunt resistance (R_sh_) was estimated from the inverse slope of the linear fit of the dark *J*–*V* curve in the vicinity of 0 V (−0.1 to +0.1 V), while the series resistance (R_s_) was extracted from the inverse slope of the linear region at high forward bias (1.5–1.7 V). Light-intensity-dependent open-circuit voltage (*V*_OC_) measurements were performed by recording the *V*_OC_ of the devices under different illumination intensities generated by the solar simulator. The ideality factor (*n*) was extracted from the slope of the *V*_OC_ versus ln­(*I*) relationship according to *V*_OC_ = (n*k*_B_ *T*/*q*)\ln­(*I*) + *C,* where *k*_B_ is the Boltzmann constant, *T* is the absolute temperature, *q* is the elementary charge, and *I* is the incident light intensity. Linear fitting of the *V*_OC_ versus ln­(*I*) plots was used to determine the ideality factor for each device.

## Supplementary Material



## Notes

Synthetic methods, ^1^H and ^13^C NMR spectra, device fabrication methods and testing, computational details, material characterization details, ATR-FTIR, scanning electron microscopy, *J*–*V* and EQE curves, transient photovoltage, light-intensity dependence, transient photocurrent, and light stability measurements (PDF)
