# Supplementary material for: Triarylamine-Modified Phenothiazine Small Molecules as Hole-Transporting Materials in Wide-Band-Gap Perovskite Solar Cells
Source: ACS Appl Mater Interfaces. 2026 Jun 9;18(24):33826–39. doi: 10.1021/acsami.6c03041 (PMC13307074; doi:10.1021/acsami.6c03041)
Supplement: Supplementary file 1 [file am6c03041_si_001.pdf]

Supporting Information

Triarylamine modified phenothiazine small molecules as hole transporting materials in wide band gap Perovskite Solar Cells

Daniel Augusto Machado de Alencar,[a,] Jessica Barichello,[b,] Raffaele Borrelli,[c] Pierluigi Quagliotto,[a] Francesca Brunetti,[d] Matteo Bonomo[a,e,f,*] Fabio Matteocci,[d,*] Aldo di Carlo,[b,d] Claudia Barolo,[a,f,g].

[a] Department of Chemistry and NIS Interdepartmental Center, University of Turin, 10135, Turin, Italy;

[b] CNR-ISM, Istituto di Struttura della Materia, Consiglio Nazionale delle Ricerche, 00133, Rome, Italy

[c] Department of Agricultural, Forest and Food Sciences, University of Turin, Grugliasco, 10095, Turin, Italy;

[d] CHOSE, Department of Electronic Engineering, University of Rome, Tor Vergata, 00133, Rome, Italy;

[e] Department of Basic and Applied Sciences for Engineering (SBAI), Sapienza University of Rome, 00161, Rome, Italy;

[f] National Interuniversity Consortium of Materials Science and Technology (INSTM), 50121, Florence, Italy;

[g] Istituto di Scienza, Tecnologia e Sostenibilità per lo sviluppo dei Materiali Ceramici (ISSMC-CNR), 48018, Faenza, Italy

Corresponding Authors: matteo.bonomo@uniroma1.it, fabio.matteocci@uniroma2.it

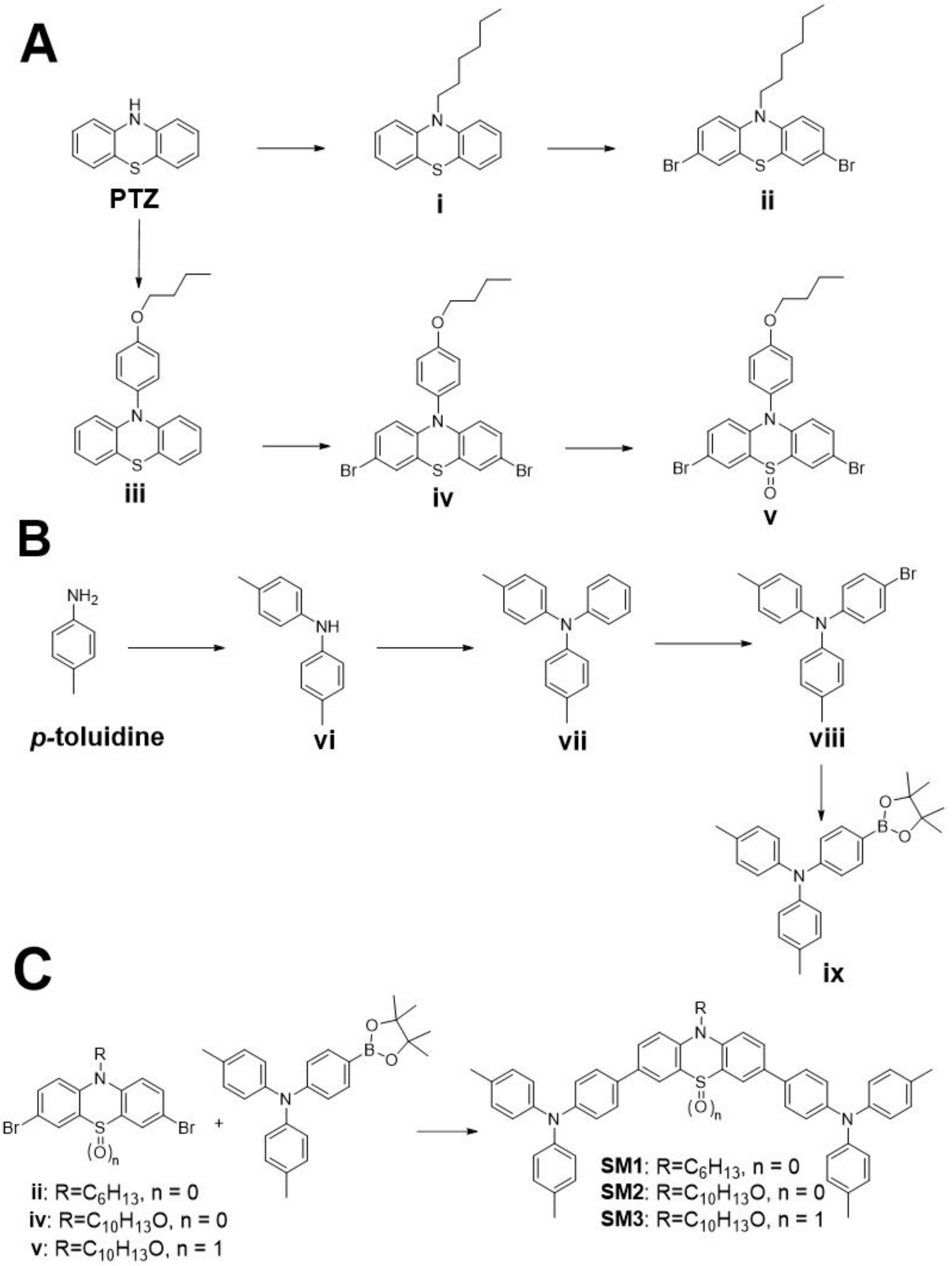

Scheme S1 - Synthesis routes for obtaining the intermediates and the HTMs (SM1-3) **A** (i) $C_6H_{13}I$, NaH, DMF, 0 ºC, 5 hrs (ii) Br_2, DCM, 15 mins (iii) 1-Bromo-4-butoxybenzene, RuPhos-Pd-G2, Na^tBuO, toluene, 110 ºC, overnight (iv) Br_2, DCM, 15 mins (v) mCPBA, DCM, 0 ºC, 5 hrs **B** (vi) *p*-bromotoluene, RuPhos-Pd-G2, Na^tBuO, toluene, 110 ºC, overnight (vii) bromobenzene, RuPhos-Pd-G2, Na^tBuO, toluene, 110 ºC, overnight (viii) NBS, $CHCl_3$, 0 ºC, (ix) B_2Pin_2, $Pd(PPh_3)_2Cl_2$, dioxane, 100 ºC, overnight **C** $Pd(PPh_3)_4$, K_2CO_3, toluene, 110 ºC, 48 hours.

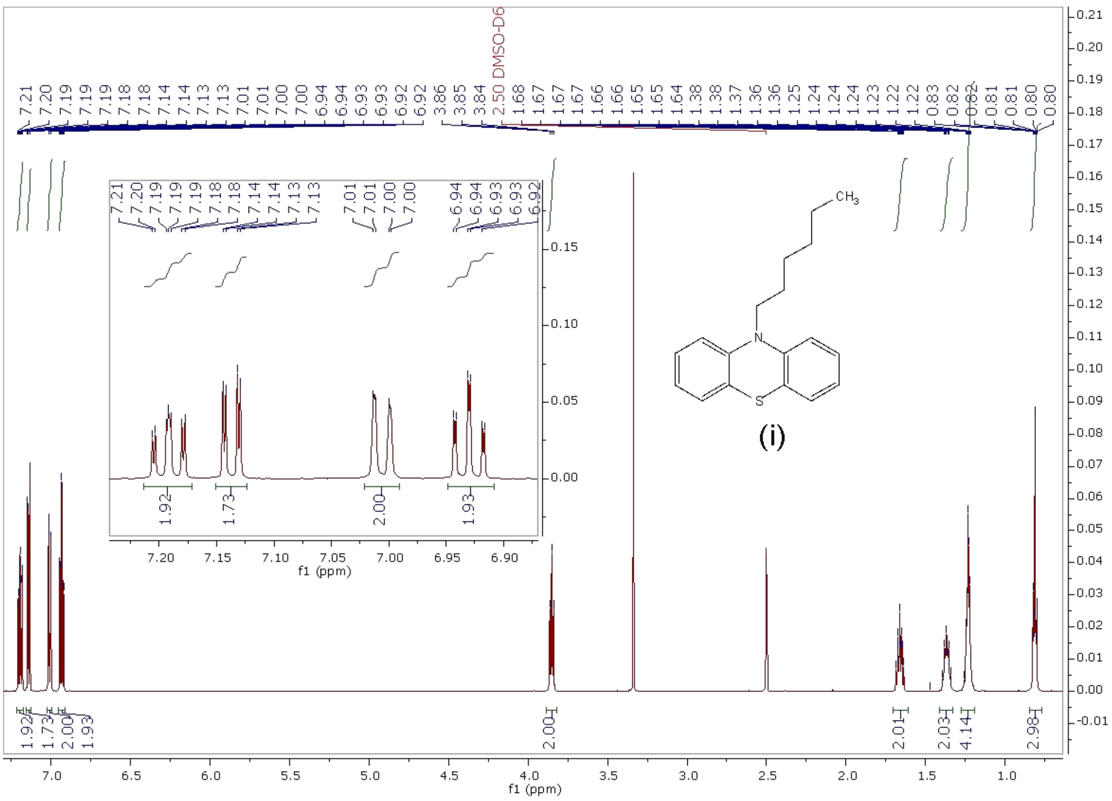

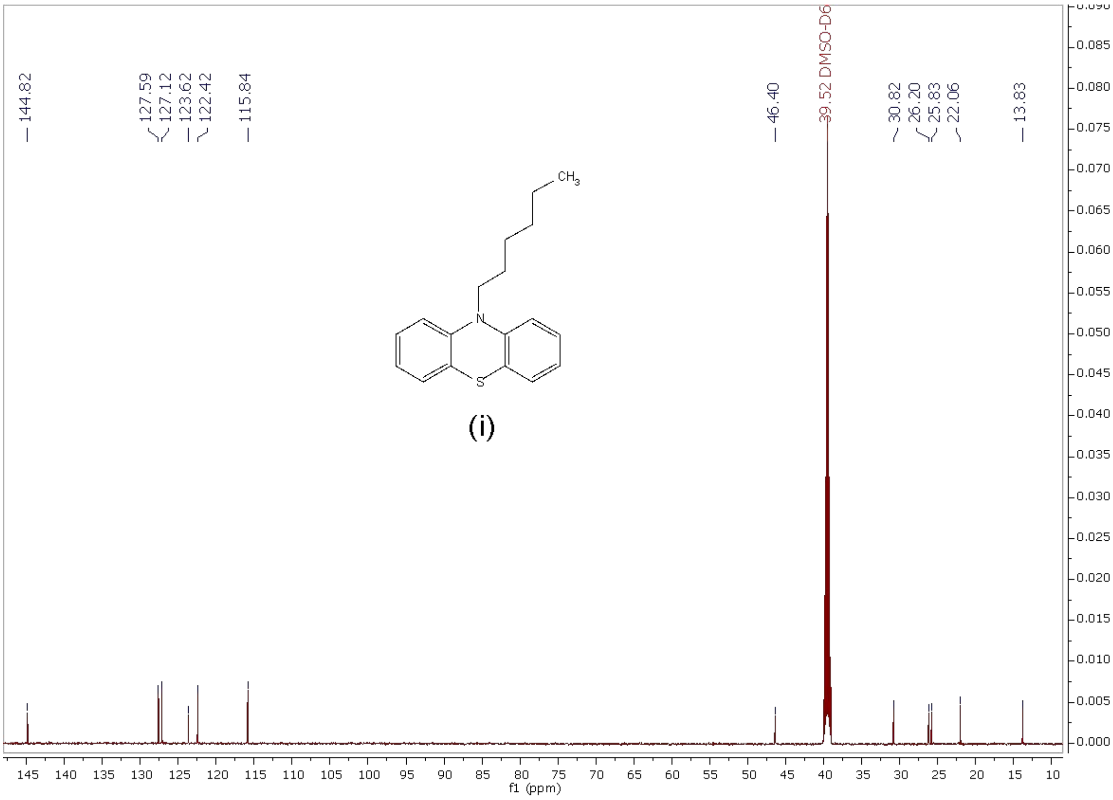

Figure S1 – ^{1}H and ^{13}C NMR spectra of intermediate *i*

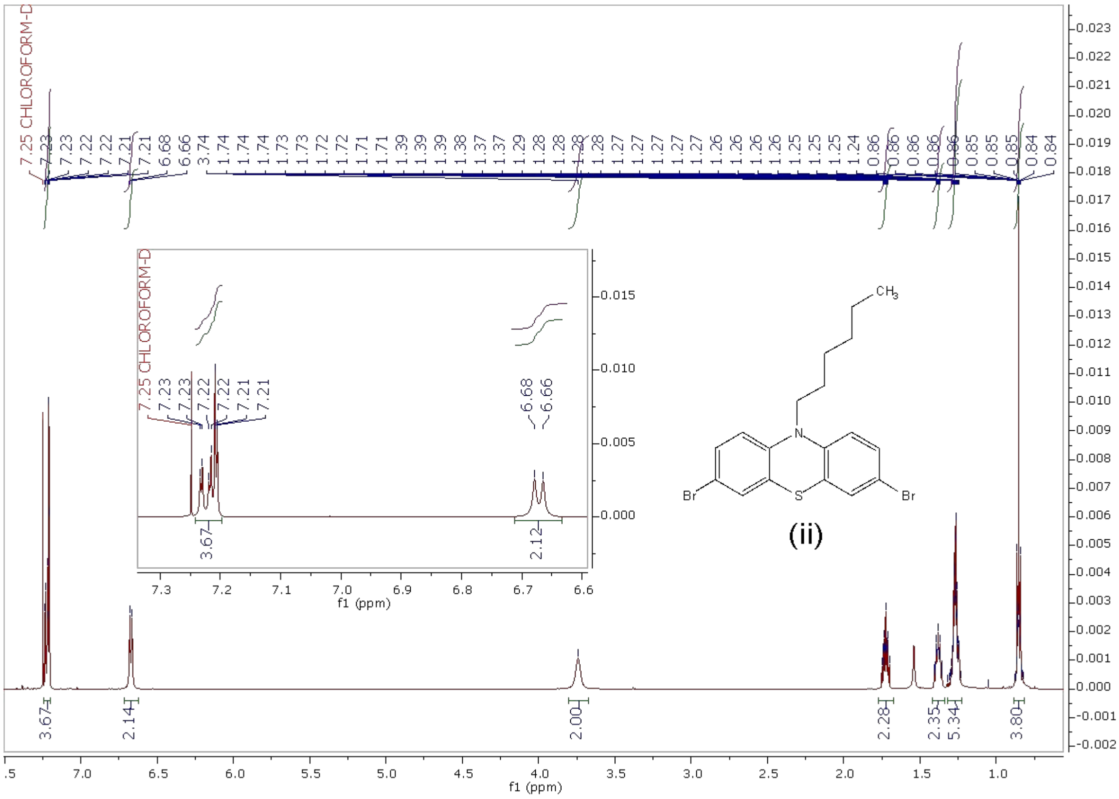

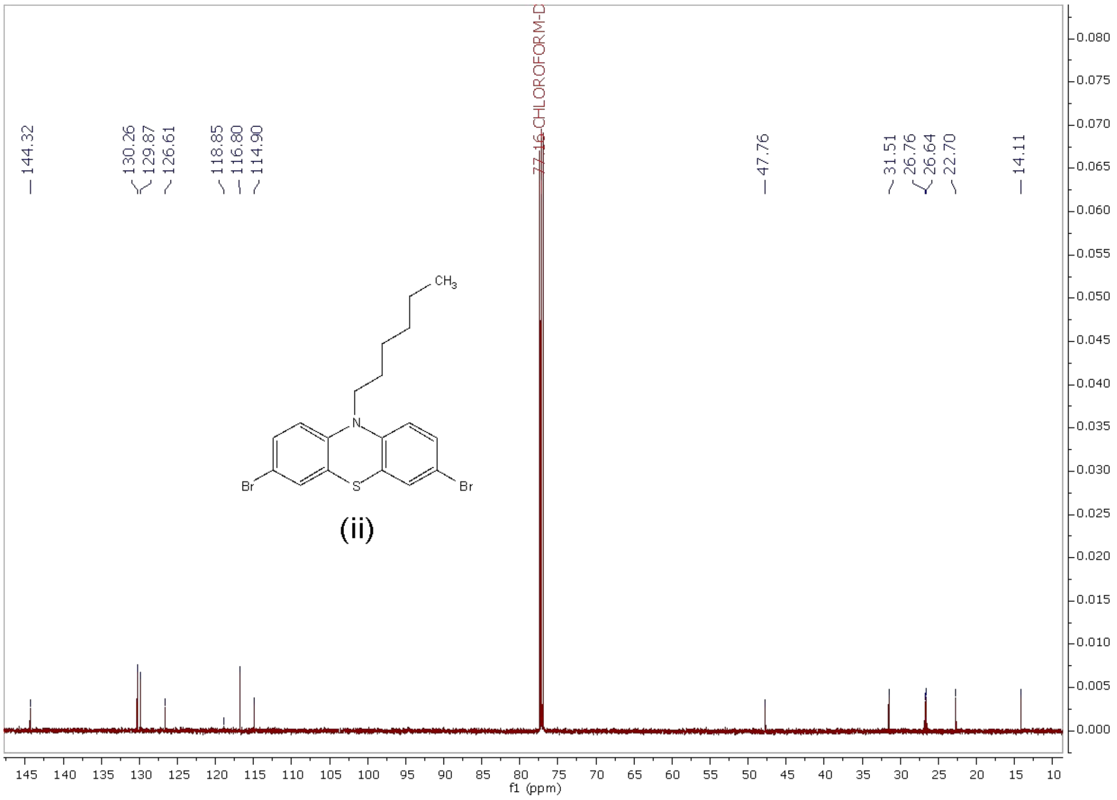

Figure S2 – ^{1}H and ^{13}C NMR spectra of intermediate *ii*

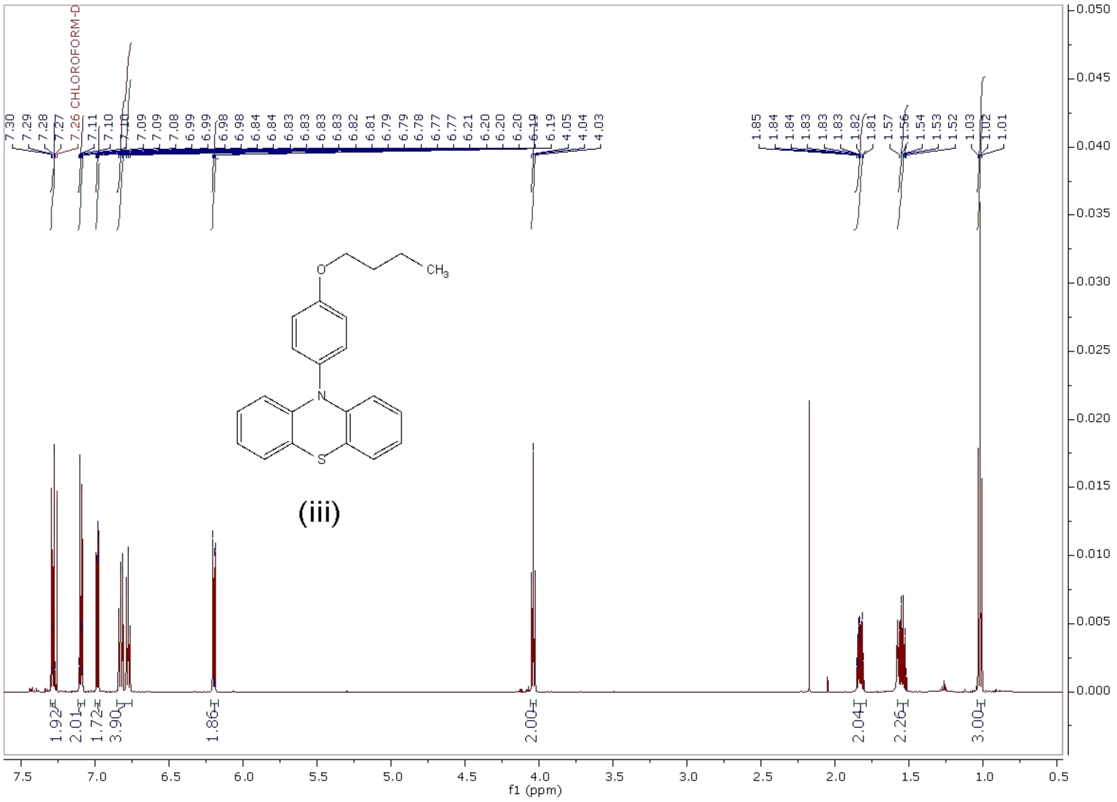

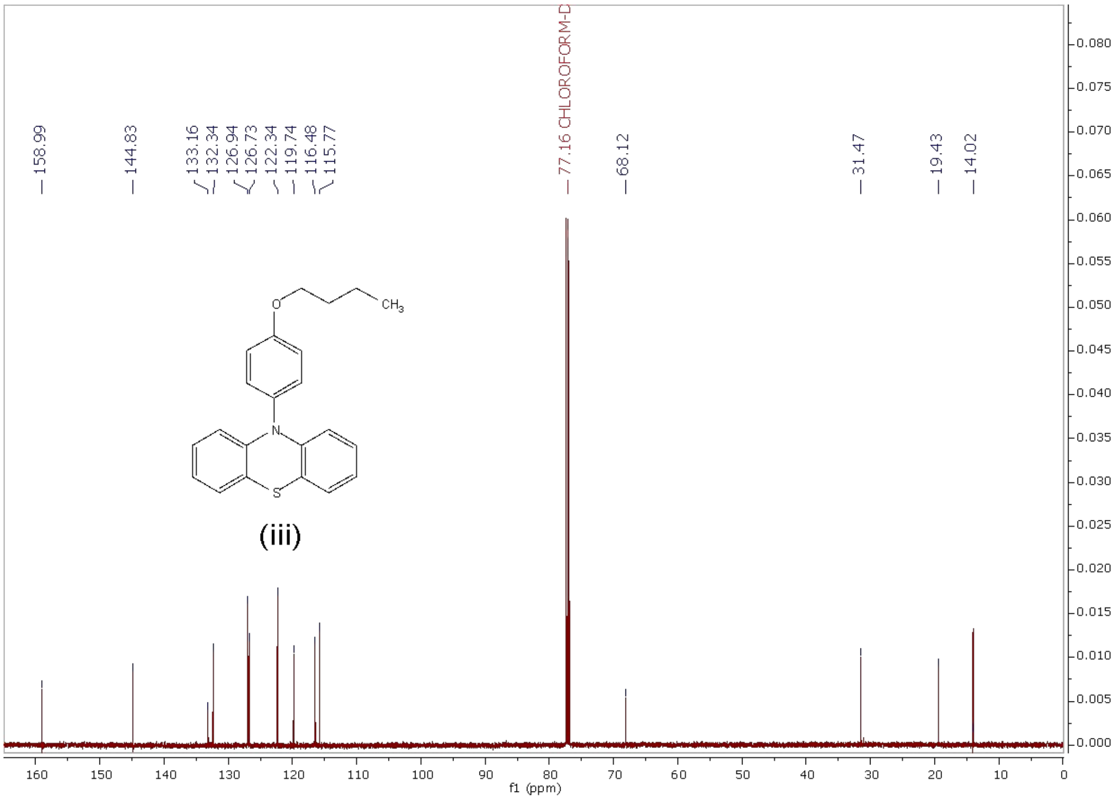

Figure S3 – ^{1}H and ^{13}C NMR spectra of intermediate *iii*

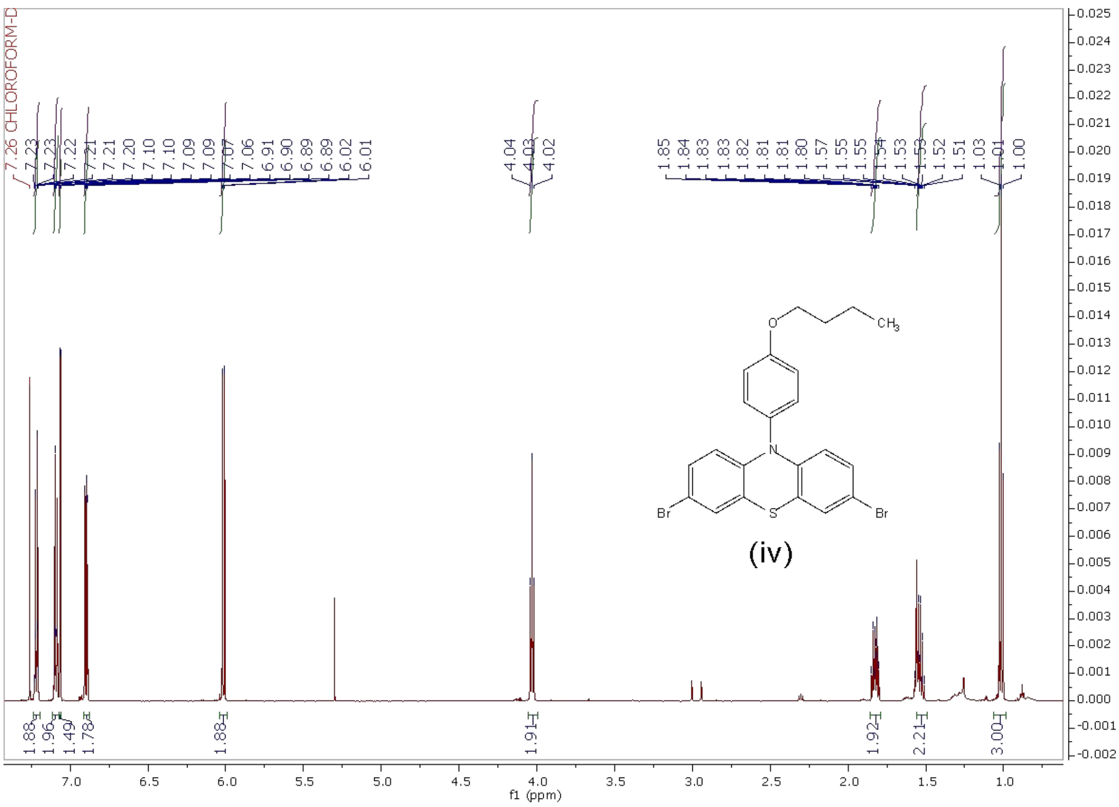

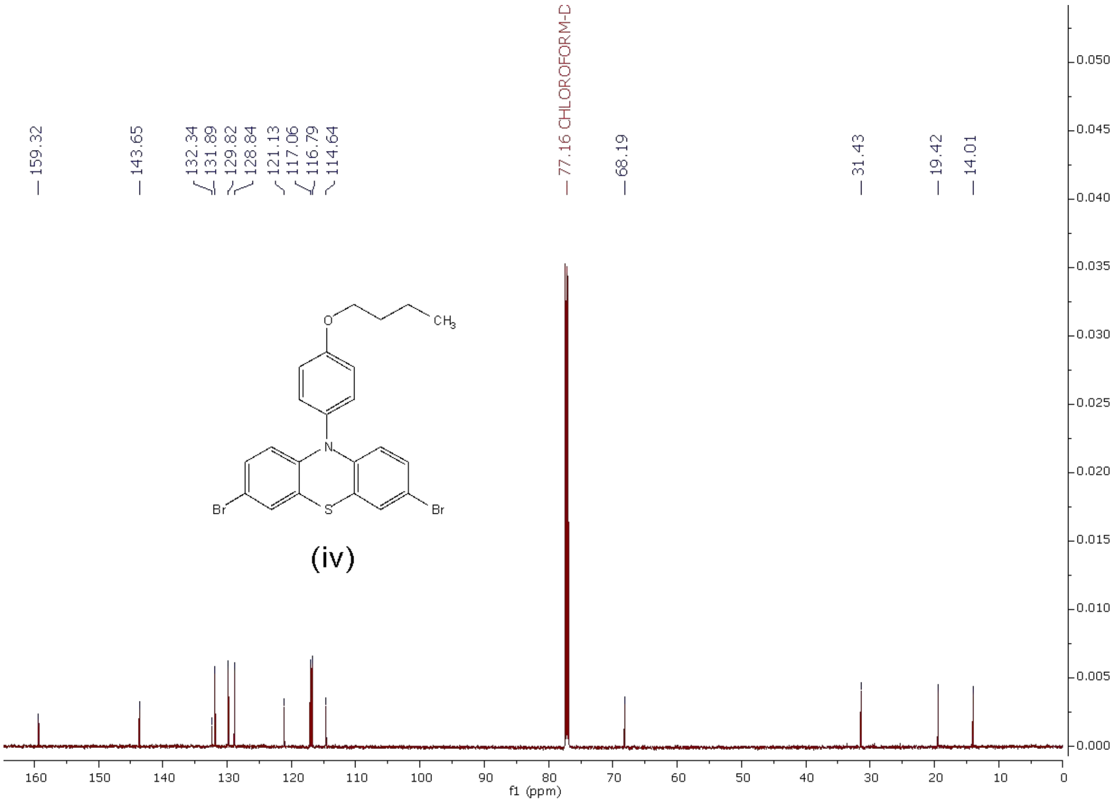

Figure S4 – ^{1}H and ^{13}C NMR spectra of intermediate iv

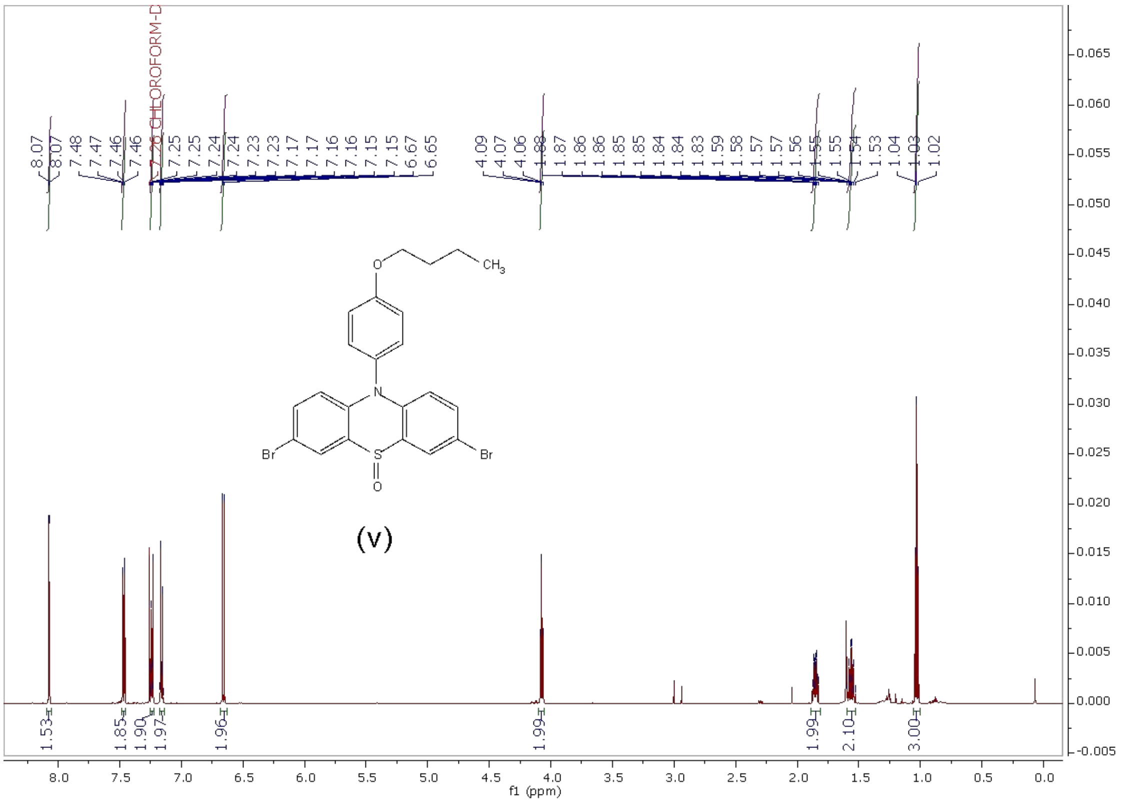

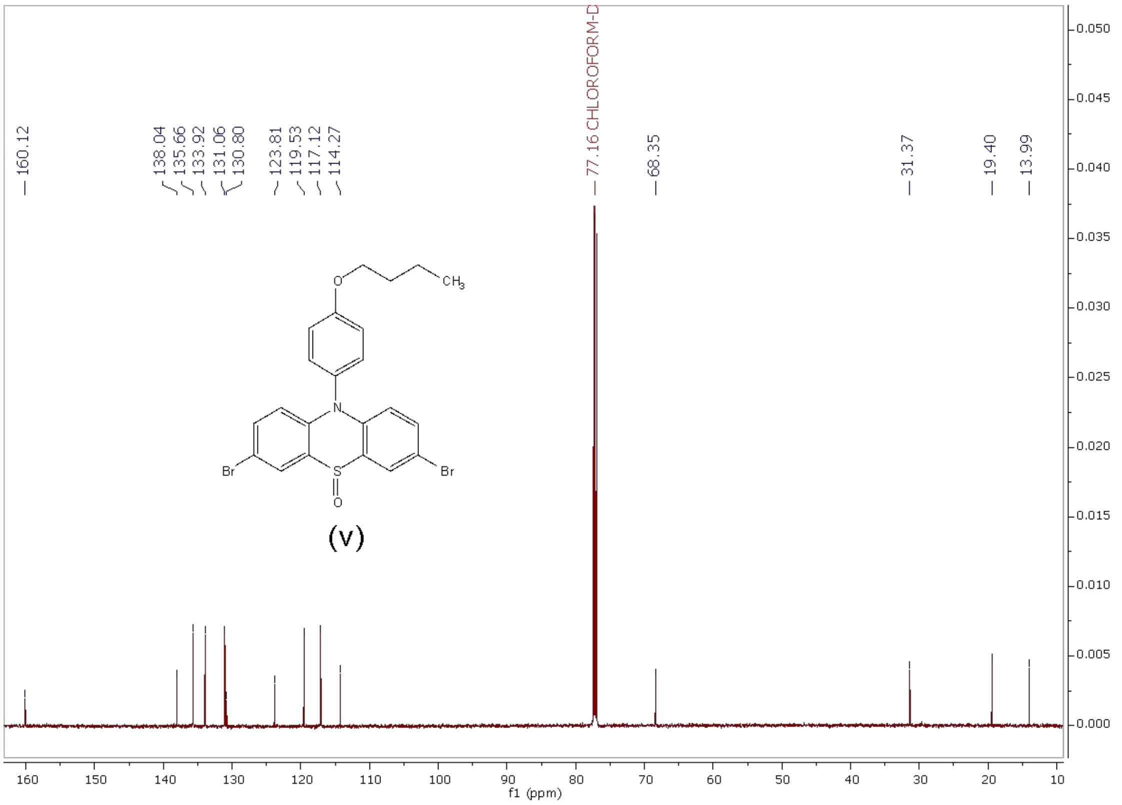

Figure S5 – ^{1}H and ^{13}C NMR spectra of intermediate v

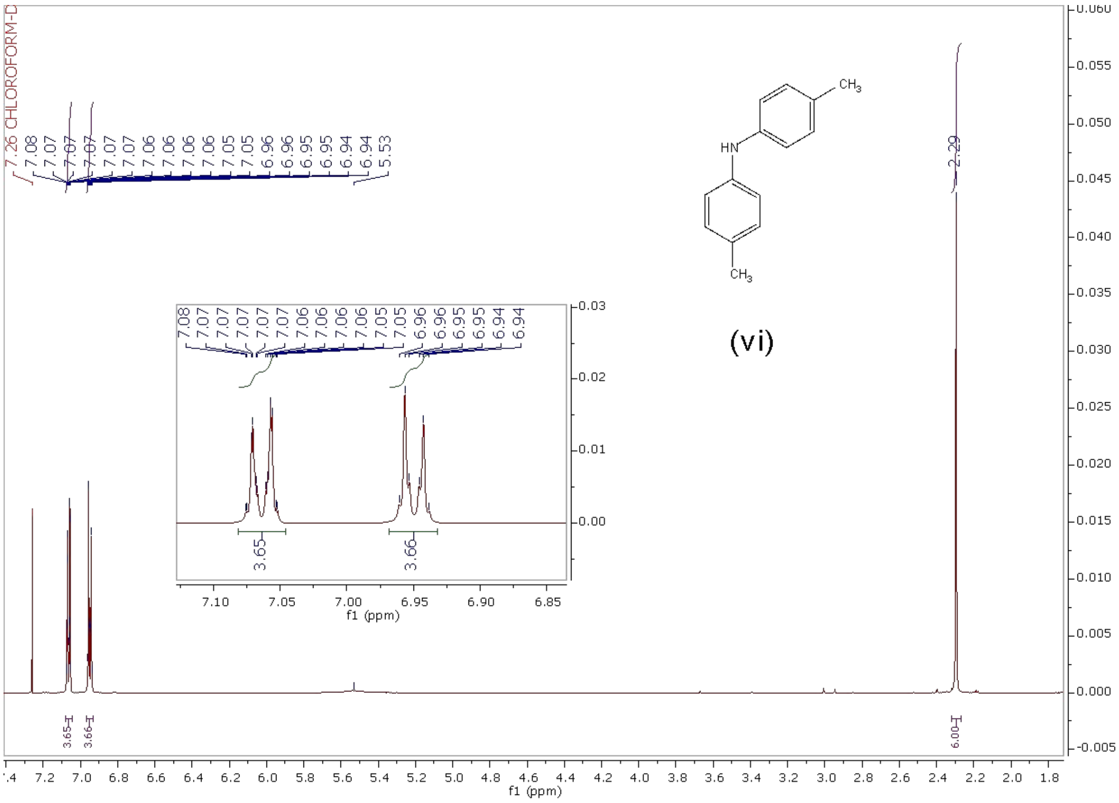

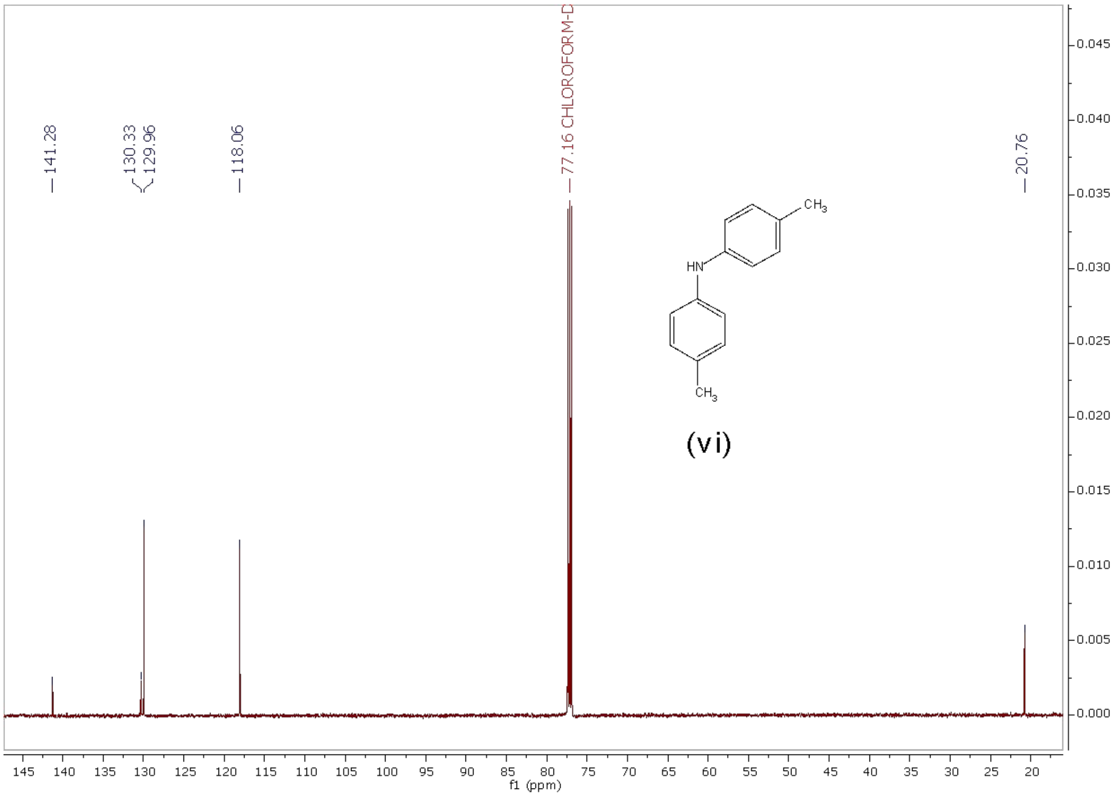

Figure S6 – ^{1}H and ^{13}C NMR spectra of intermediate vi

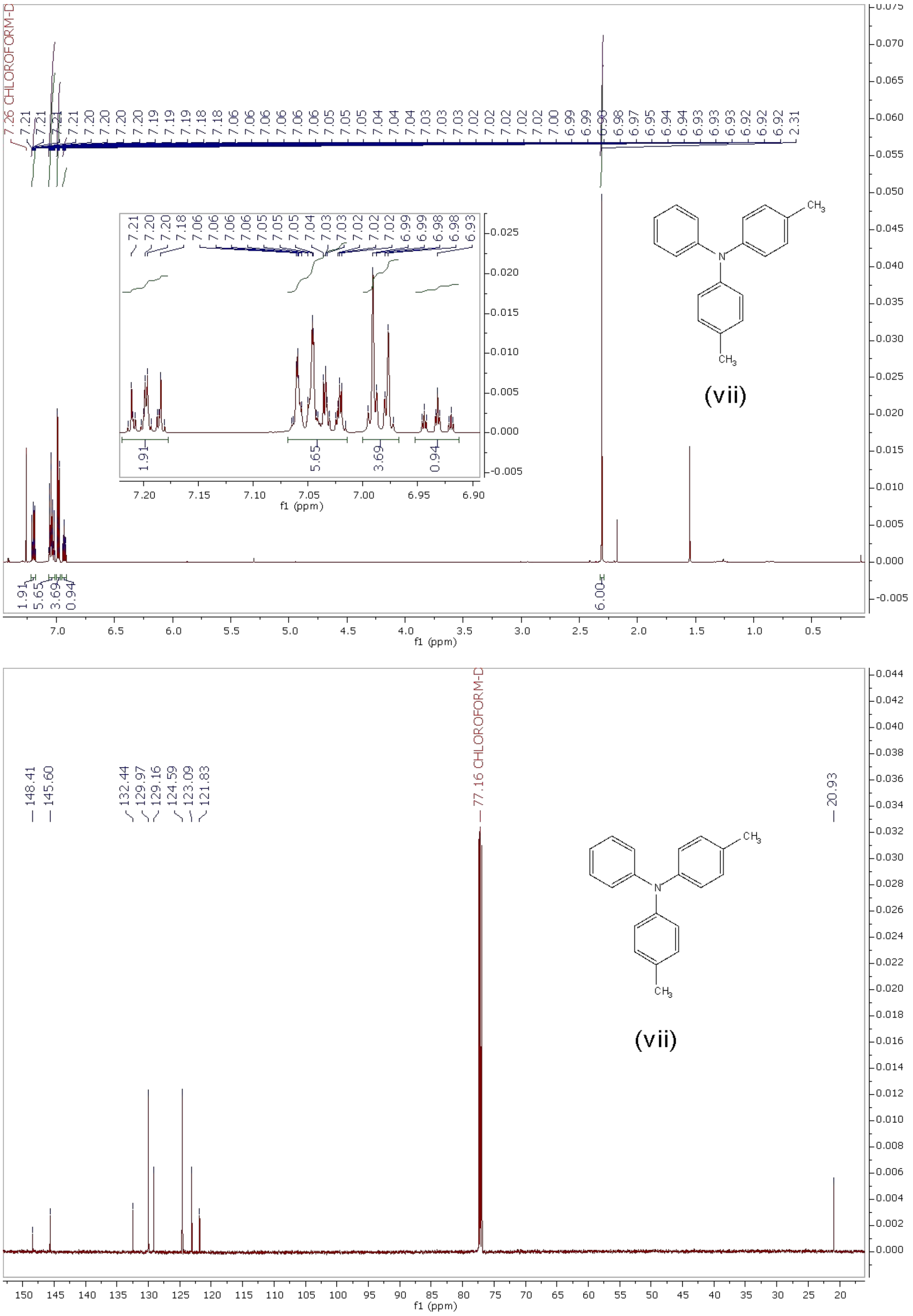

Figure S7 – ^{1}H and ^{13}C NMR spectra of intermediate vii

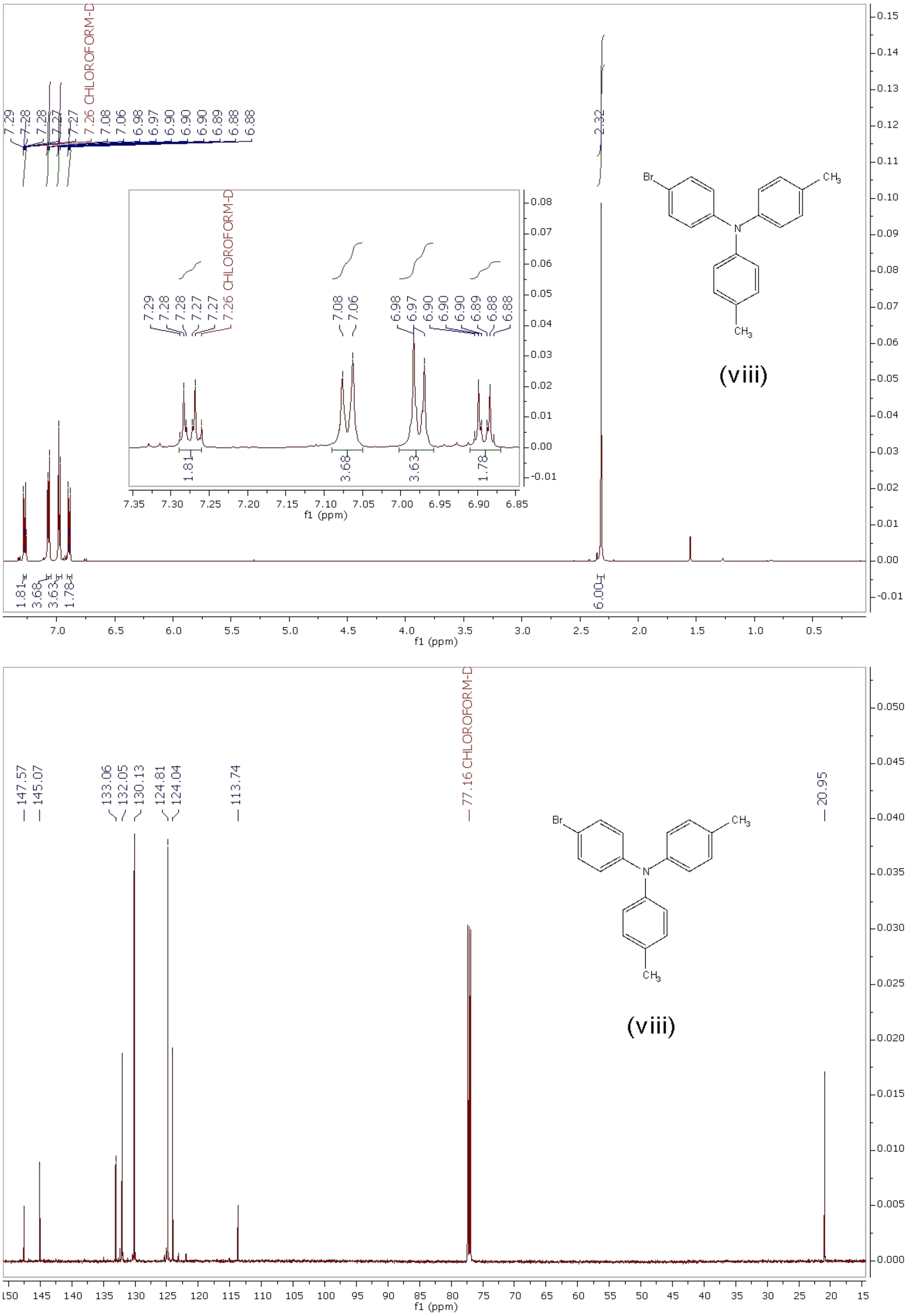

Figure S8 – ^{1}H and ^{13}C NMR spectra of intermediate viii

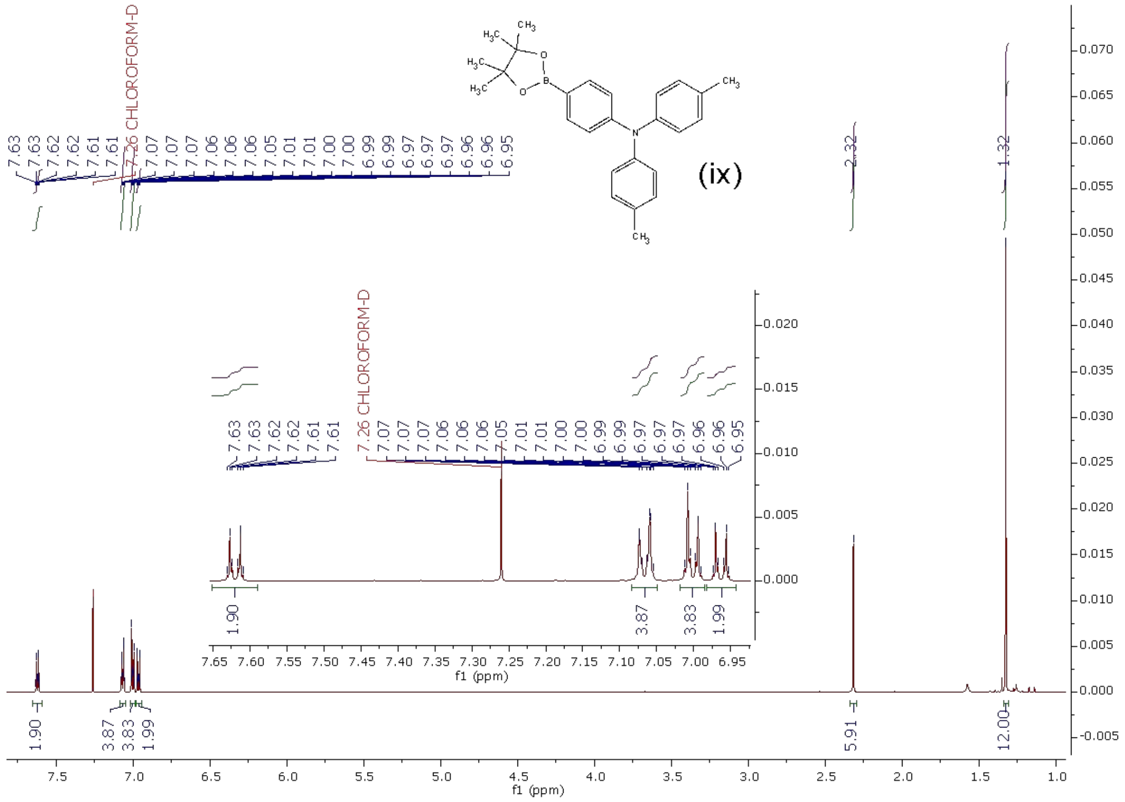

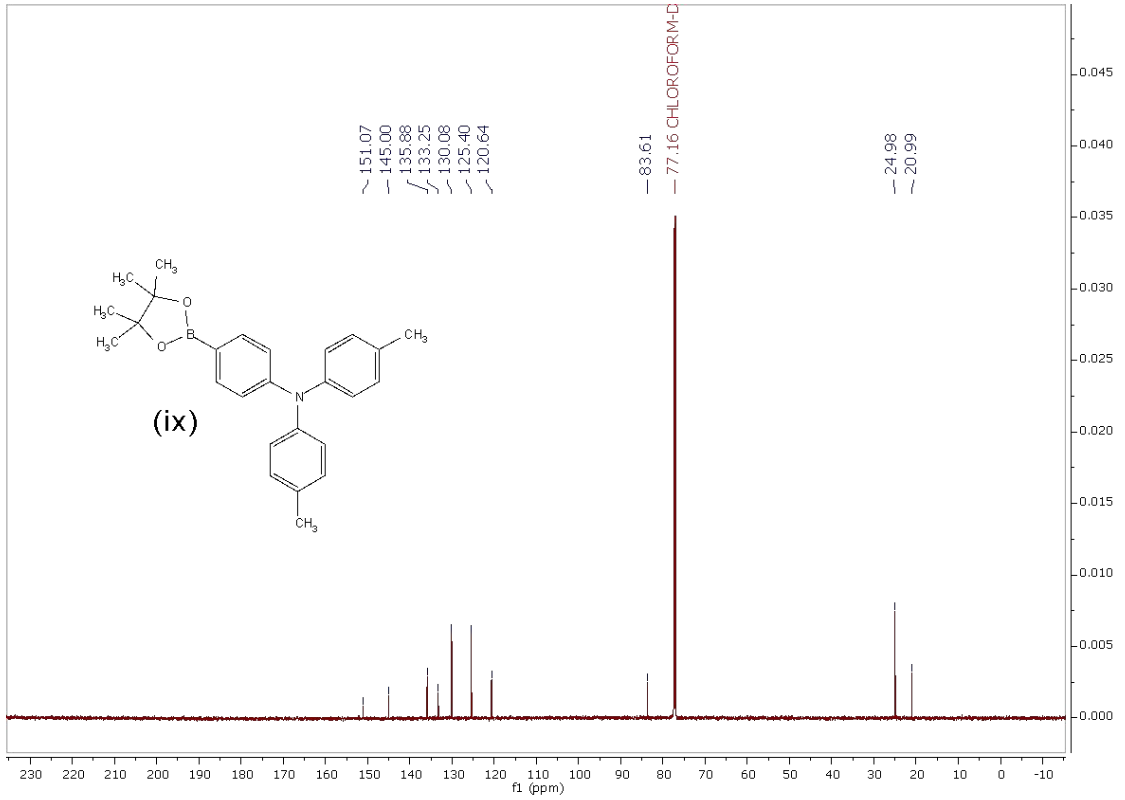

Figure S9 – ^{1}H and ^{13}C NMR spectra of intermediate ix

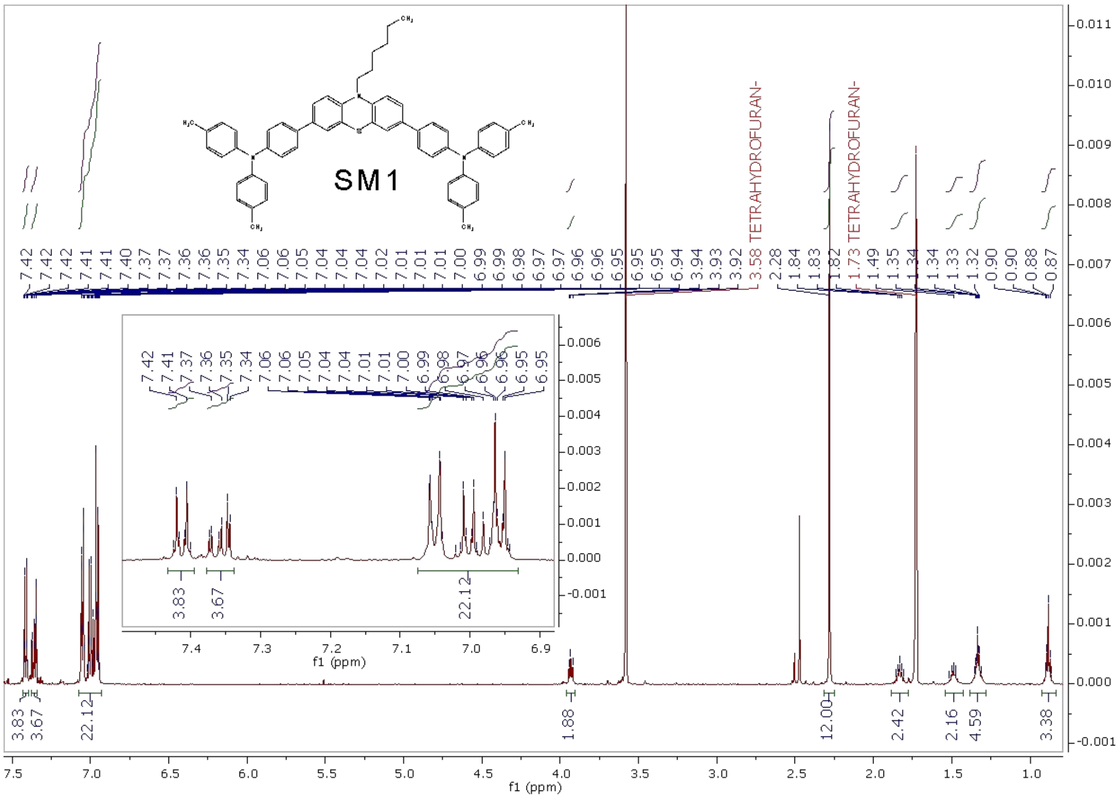

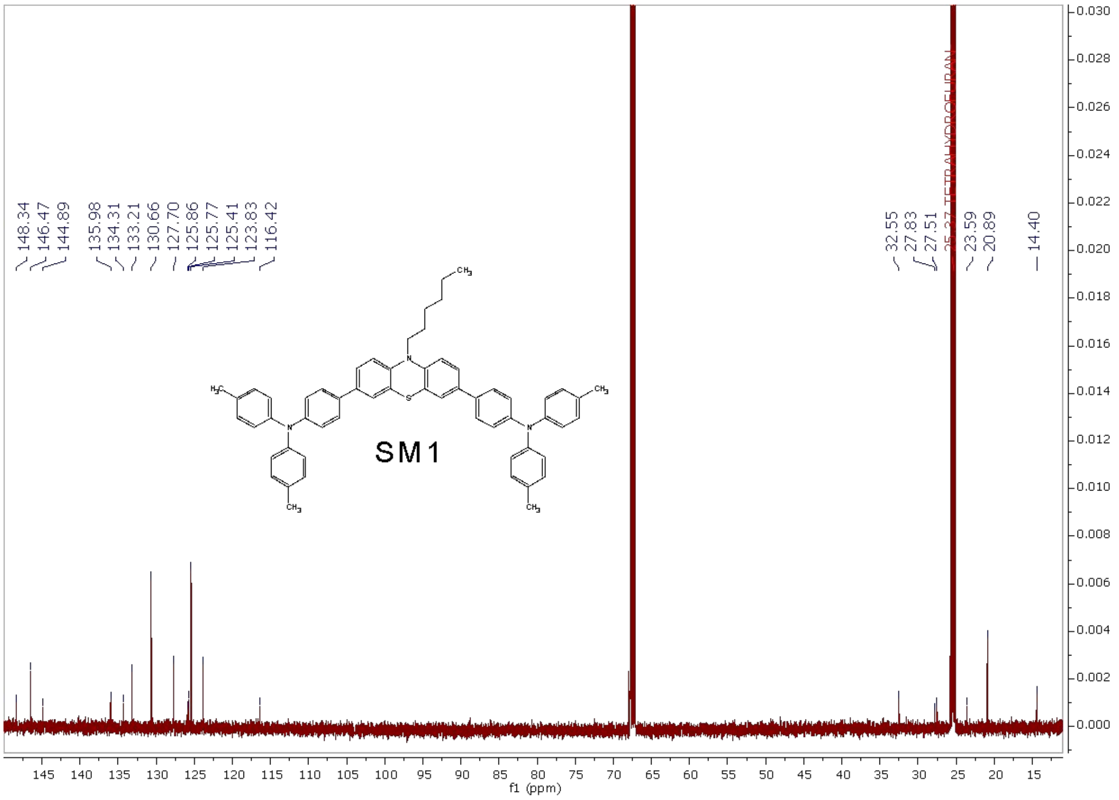

Figure S10 – ^{1}H and ^{13}C NMR spectra of SM1

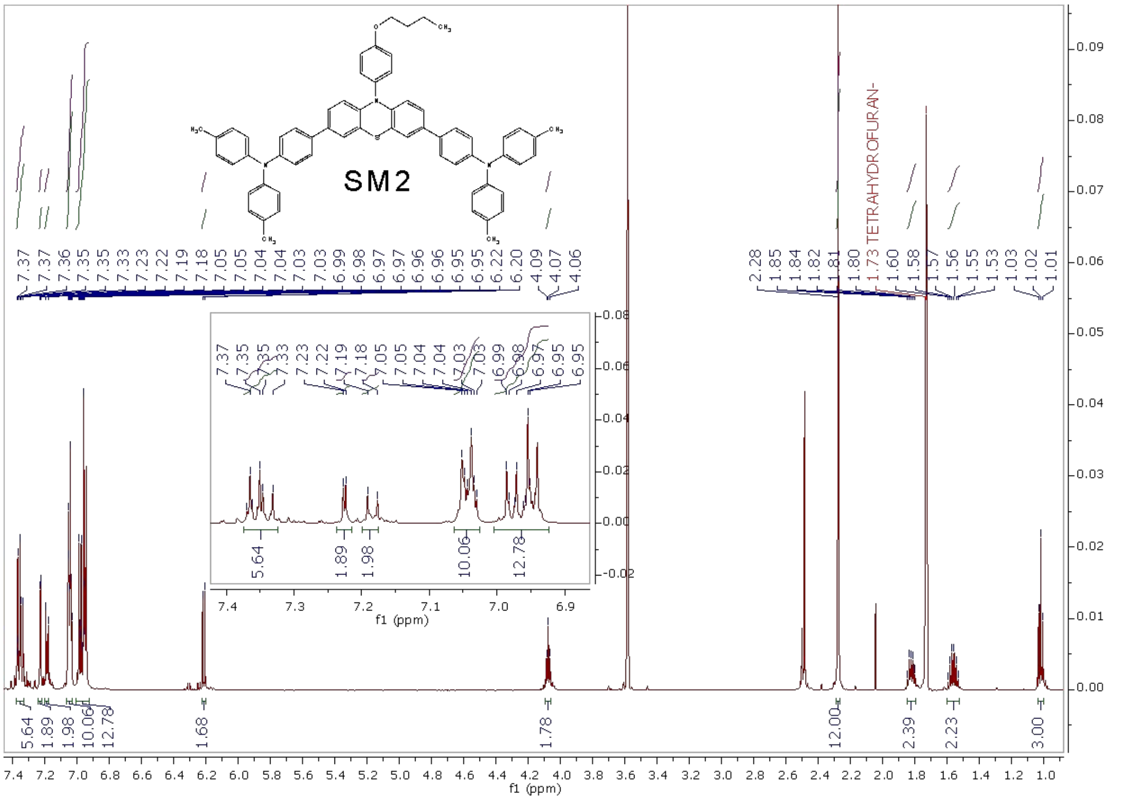

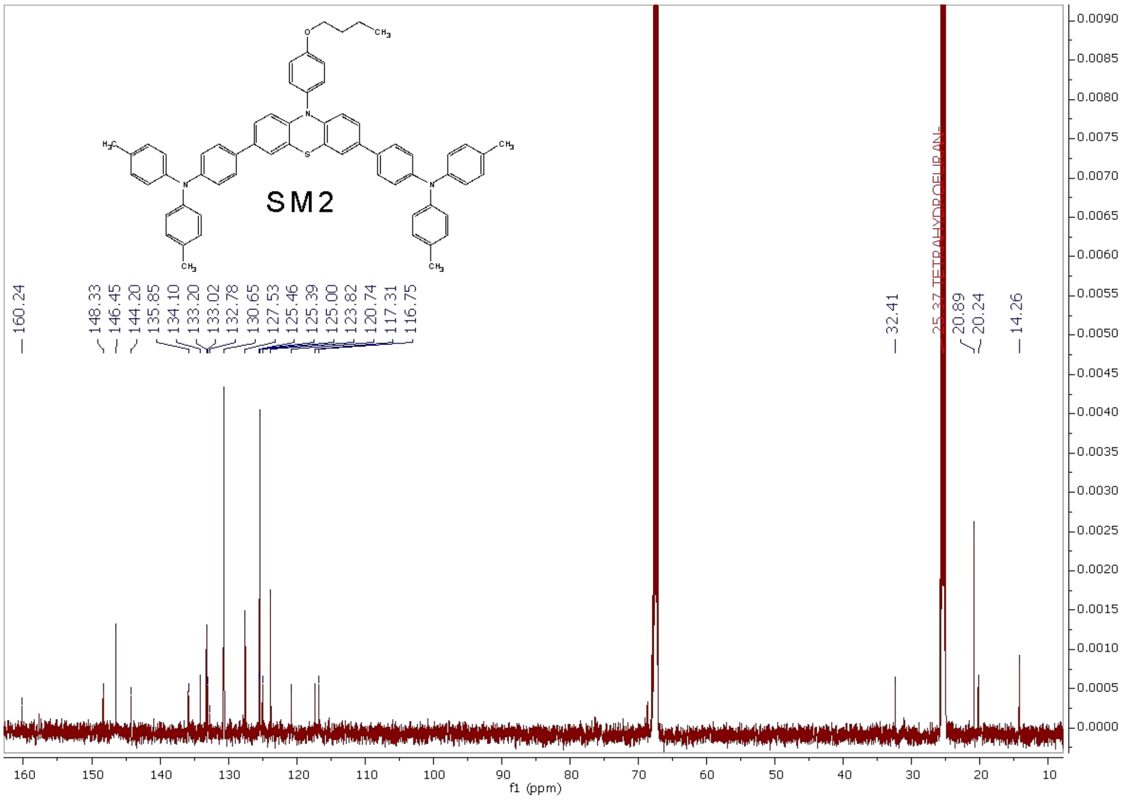

Figure S11 – ^{1}H and ^{13}C NMR spectra of SM2

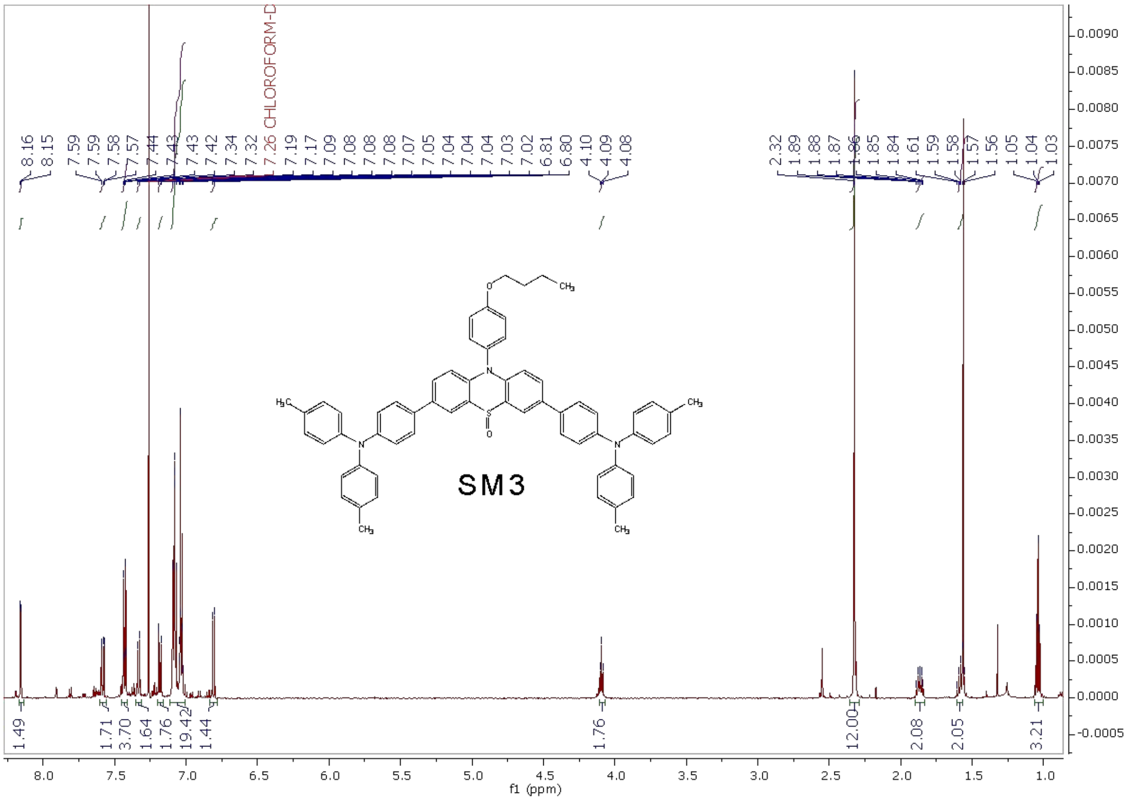

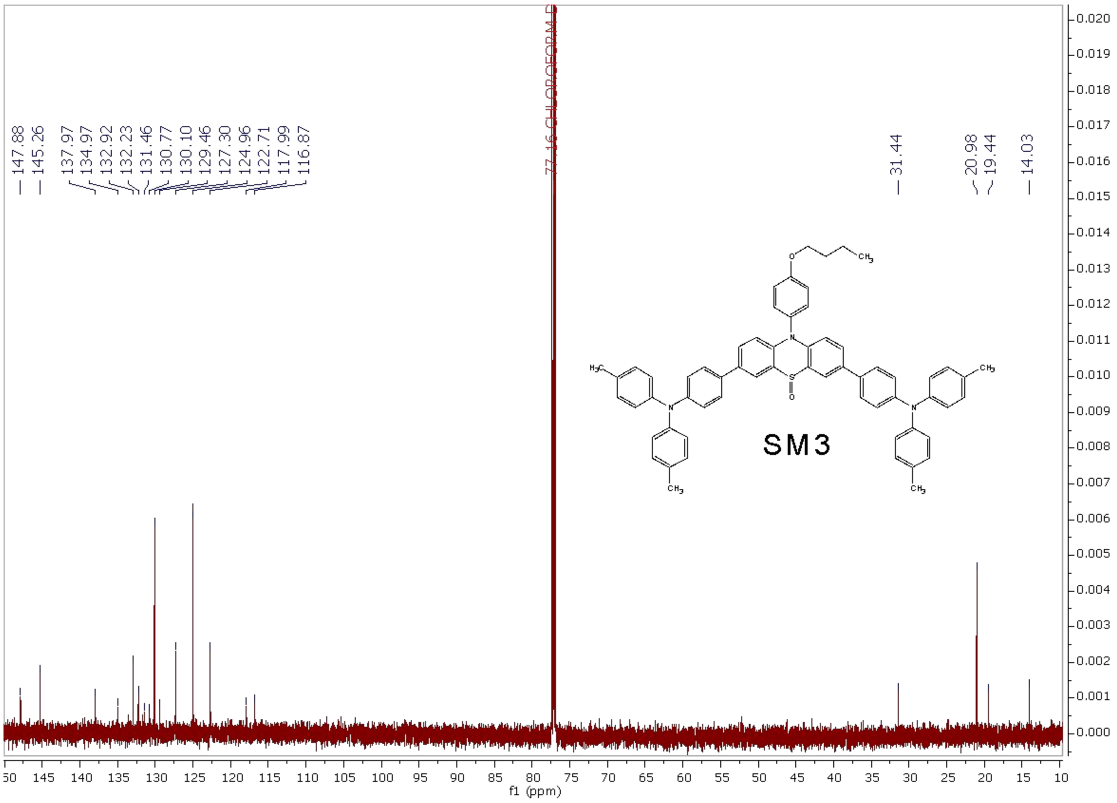

Figure S12 – ^{1}H and ^{13}C NMR spectra of SM3

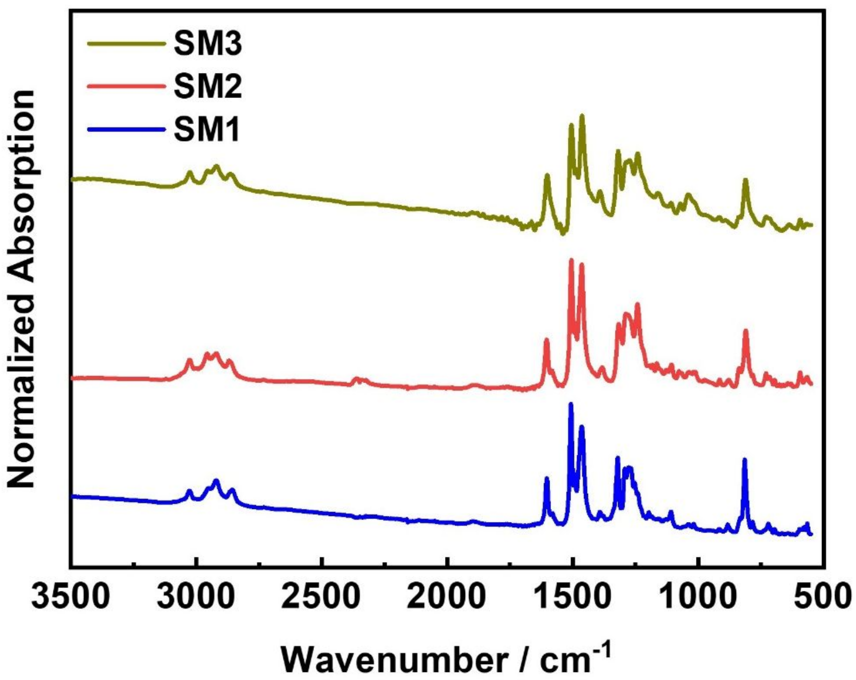

Figure S13 – ATR-FITR spectra of SM1-3

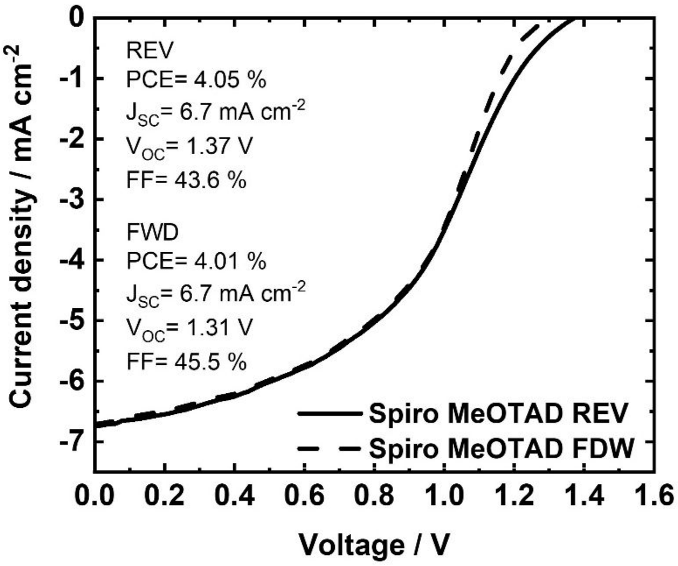

Figure S14 – Spiro-OMeTAD as HTM in semi-transparent $FaPbBr_3$ based PSC in RV and FWD scan with sputtered ITO as top electrode

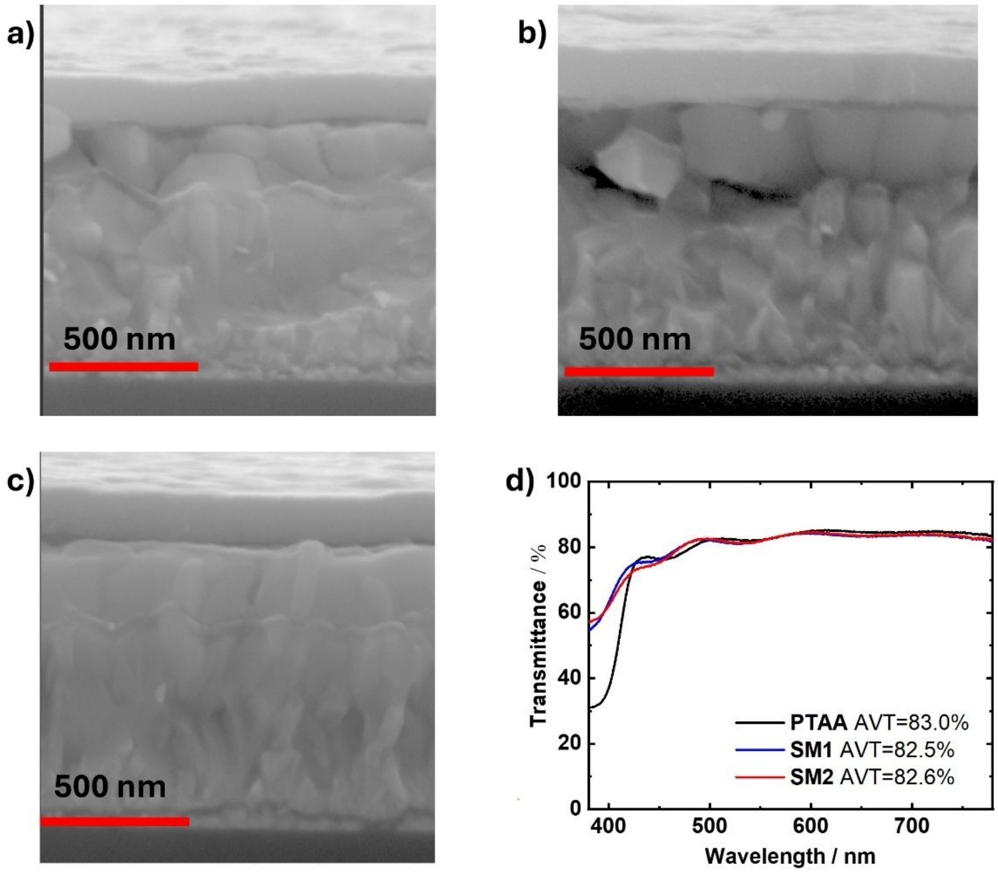

Figure S15 (A–C) Cross-sectional SEM images of semi-transparent $FAPbBr_3$ perovskite solar cells employing (A) PTAA, (B) SM1, and (C) SM2 as hole-transporting materials (HTMs), showing comparable layer thicknesses across the different devices. (D) Optical transmittance spectra of PTAA, SM1, and SM2 films deposited on FTO substrates, with average visible transmittance (AVT) values of 83.0%, 82.5%, and 82.6%, respectively, indicating similar transparency in the visible range.

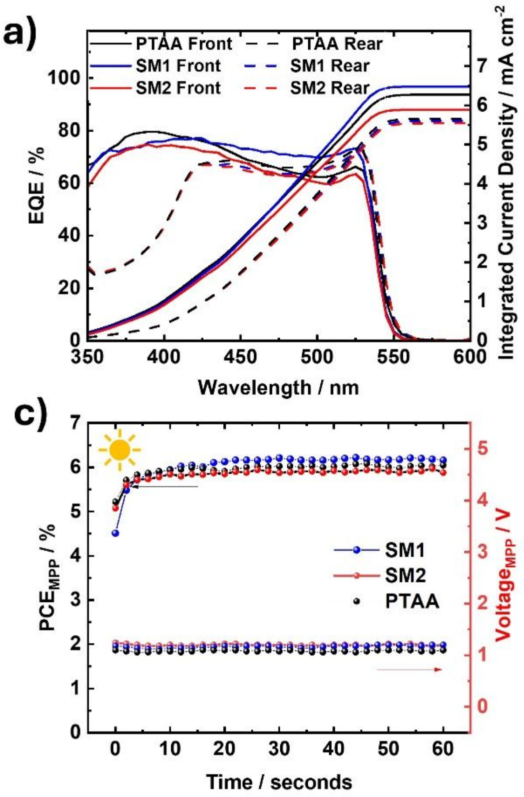

Figure S16 –(a) EQE spectra and corresponding integrated current density for semi-transparent $FAPbBr_3$ devices employing PTAA, SM1, and SM2 as hole-transporting materials, measured under front and rear illumination. (b) Optical transmittance spectra of the complete devices, highlighting the comparable transparency of all HTMs in the visible range. (c) Maximum power point tracking (MPPT) under 1 SUN illumination, showing the evolution of power conversion efficiency (PCE_{MPP}, left axis) and operating voltage (V_{MPP}, right axis) over 60 s. (d) MPPT under indoor illumination (1000 lux), demonstrating rapid stabilization and higher steady-state PCE for SM-based devices compared to PTAA.

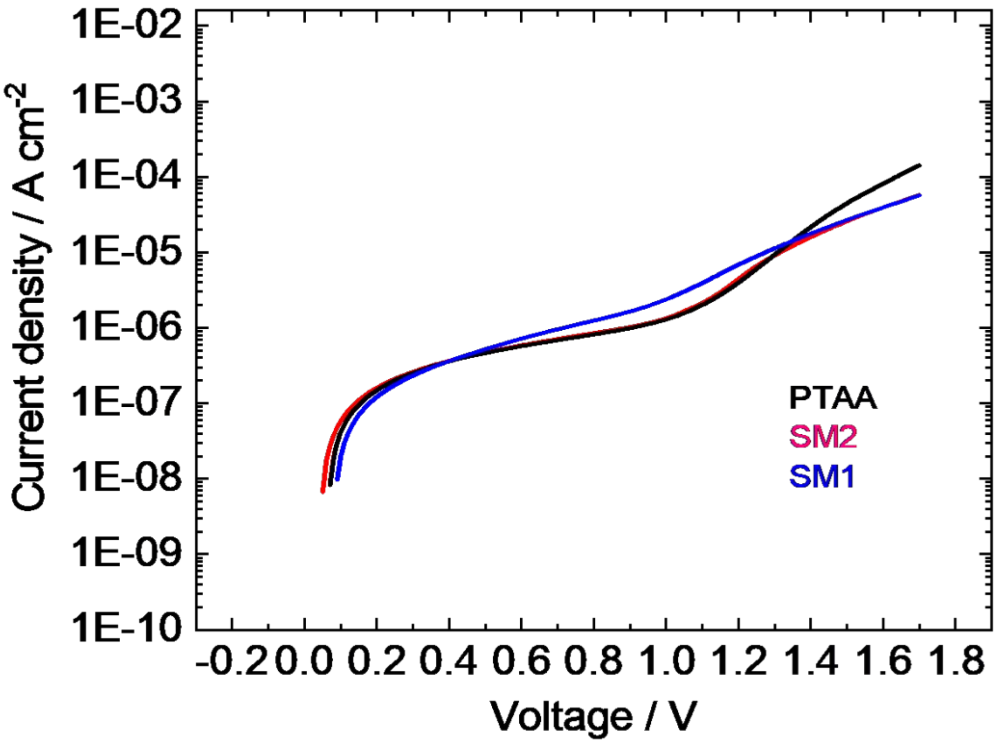

Figure S17 Dark current density–voltage (J–V) characteristics of semi-transparent $FAPbBr_3$ perovskite solar cells employing PTAA, SM1, and SM2 as hole-transporting materials.

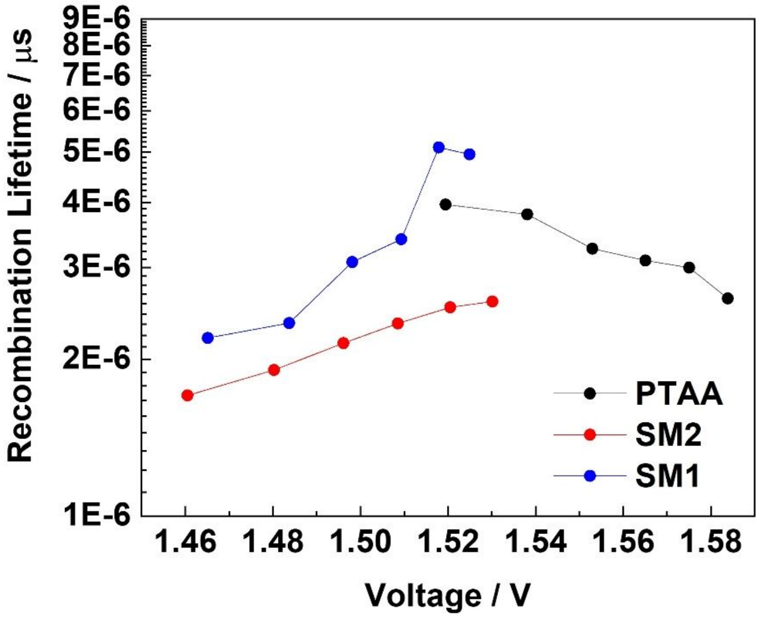

Figure S18 Recombination Time vs. Voltage extracted from TPV characterization by varying the illumination intensity of the pulsed light.

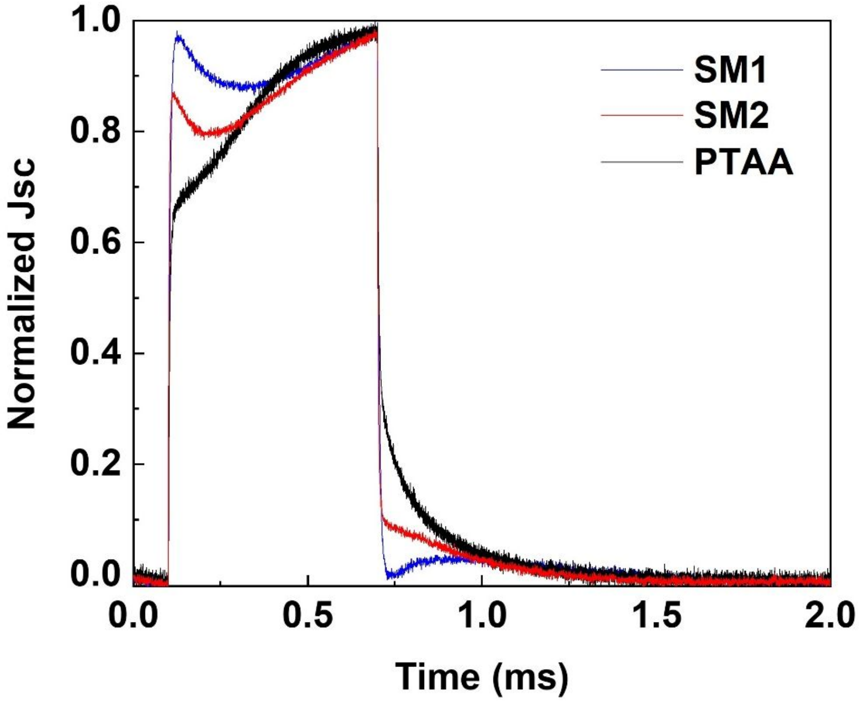

Figure S19 Normalized Photocurrent of the complete devices by varying the HTM at low illumination.

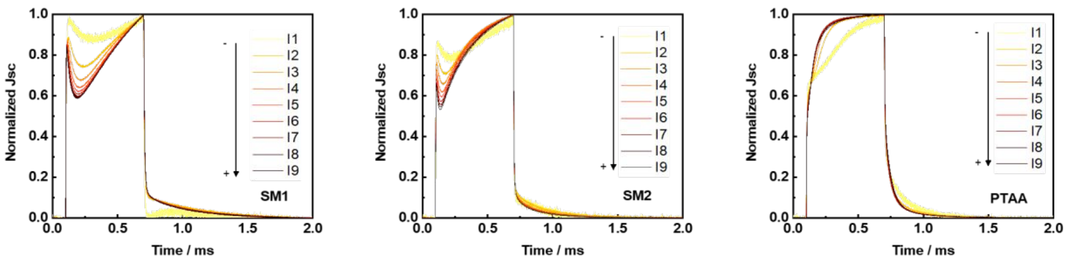

Figure S20 Normalized Jsc current density profiles measured by intensity-dependent TPC characterization varying the HTM.

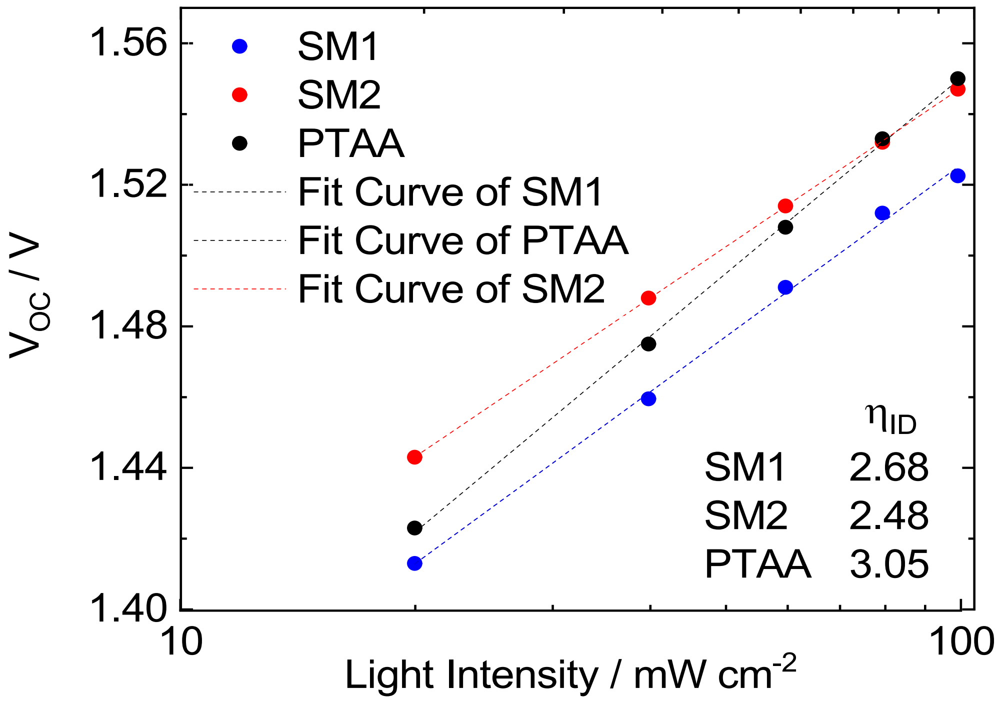

Figure S21 Open-circuit voltage (V_{OC}) as a function of light intensity for $FAPbBr_3$ perovskite solar cells employing PTAA, SM1, and SM2 as hole-transporting materials. The dashed lines represent linear fits of V_{OC} versus ln(I), from which the ideality factors (n) were extracted.

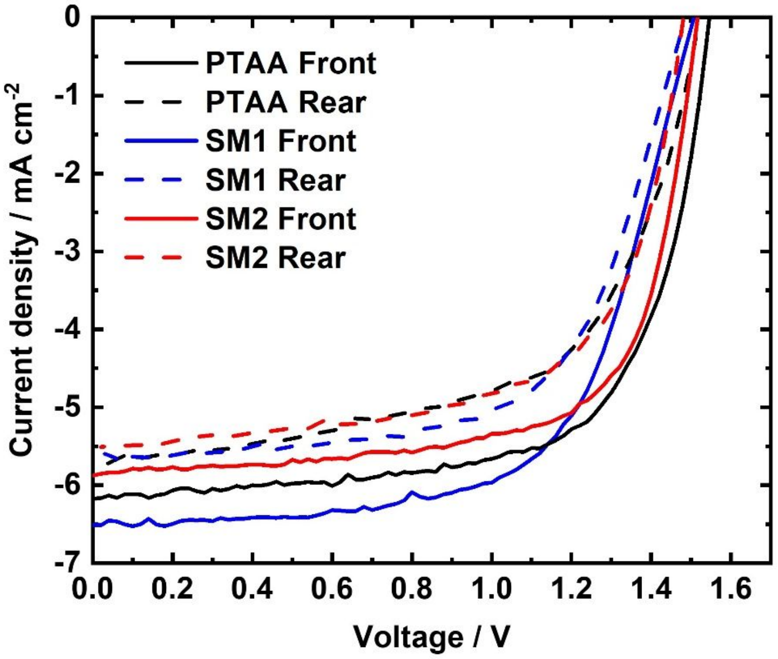

Figure S22 J–V characteristics of semi-transparent $FAPbBr_3$ perovskite solar cells employing PTAA, SM1, and SM2 as hole-transporting materials, measured under front (solid lines) and rear (dashed lines) illumination at 1 SUN.

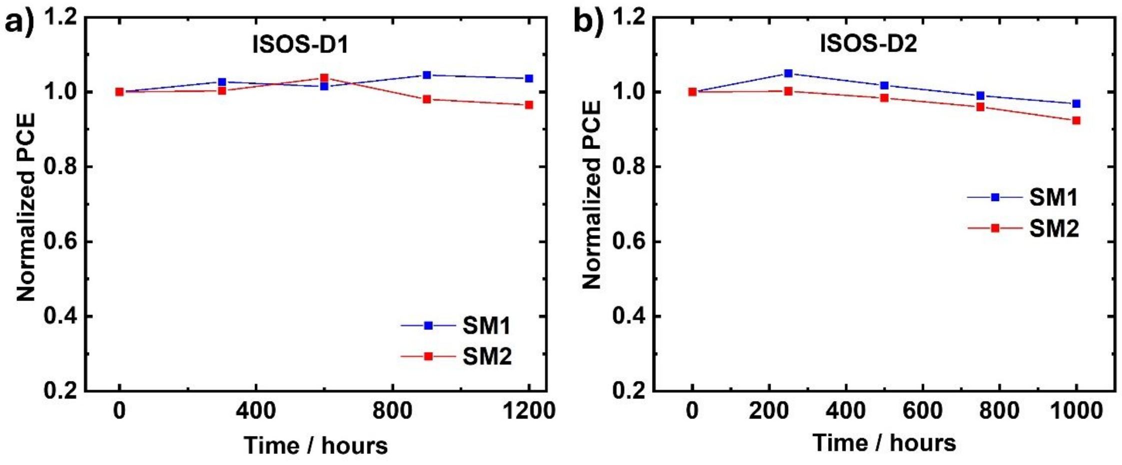

Figure S23 Stability assessment of semi-transparent $FAPbBr_3$ perovskite solar cells employing SM1 and SM2 as hole-transporting materials (HTMs). (A) ISOS-D1 test, showing normalized PCE as a function of time under ambient environmental conditions, with negligible performance degradation over 1200 h. (B) ISOS-D2 test, showing normalized PCE under dark storage at 85 °C, where only a slight decrease in efficiency is observed after 1000 h, indicating good thermal stability of the small-molecule HTMs.

Table S1 - Calculated oxidation potentials in vacuum for the SM1–SM3 scaffolds bearing two TPA units (2TPA), one TPA unit (1TPA), or no TPA substituent (no-TPA).

Scaffolds	E in Vacuum [eV]
**SM1**-2TPA	-4.95
**SM1**-1TPA	-4.96
**SM1**-no-TPA	-5.39
**SM2**-2TPA	-4.99
**SM2**-1TPA	-5.02
**SM2**-no-TPA	-5.18
**SM3**-2TPA	-5.10
**SM3**-1TPA	-5.11
**SM3**-no-TPA	-5.87

Table S2 Shunt resistance (R_{sh}) and series resistance (R_s) extracted from dark J–V measurements of perovskite solar cells employing PTAA, SM1, and SM2 as hole-transporting materials.

	R_{sh} [kΩ·cm²]	R_s [Ω·cm²]
**PTAA**	53	208.4
**SM1**	100	667
**SM2**	72.9	635.6

Table S3 - Photovoltaic parameters of the best-performing semi-transparent $FAPbBr_3$ PSC measured under front and rear illumination at 1 SUN, employing PTAA, SM1, and SM2 as HTM, together with the corresponding bifaciality factors (BF).

		V_{OC} [V]	FF [%]	J_{SC} [mA/cm²]	PCE [%]	BFF [%]
**PTAA**	**FRONT**	1.54	65.4	6.17	6.39	0.81
	**REAR**	1.52	59.5	5.73	5.18	
**SM2**	**FRONT**	1.51	68.4	5.87	6.06	0.82
	**REAR**	1.48	62.9	5.63	5.19	
**SM1**	**FRONT**	1.50	63.8	6.51	6.26	0.84
	**REAR**	1.48	63.7	5.52	5.26	
